# Cellulose and wood derived activated carbons from *Pinus sylvestris*: comparative performance in methylene blue adsorption

**DOI:** 10.1039/d5ra08022c

**Published:** 2025-12-12

**Authors:** Nadia Anter, Abdellah Hannioui, Abdelouahid Medaghri-Alaoui, Mohssine Ghazoui

**Affiliations:** a Molecular Chemistry, Materials and Catalysis Laboratory, Faculty of Science and Technology, Sultan Moulay Slimane University 23000 Béni-Mellal Morocco; b Environmental, Ecological and Agro-Industrial Engineering Laboratory, Faculty of Science and Technology, Sultan Moulay Slimane University Béni-Mellal BP 592 Morocco

## Abstract

This work describes the preparation of eco-friendly activated carbons from redwood (*Pinus sylvestris* L.) sawdust and cellulose extracted from the same source, activated with H_3_PO_4_ under optimized carbonization conditions. The materials were comprehensively characterized by FTIR, SEM, TGA, XRD, and BET analyses, revealing superior textural uniformity and surface functionality for the cellulose-derived carbon (AC-CRW) compared with its wood-based counterpart (AC-RW). Adsorption experiments demonstrated consistently higher methylene blue uptake by AC-CRW, with the Langmuir isotherm and pseudo-first-order kinetics providing the best fit. Under optimal conditions (24 h impregnation, 1 : 2 impregnation ratio, 650 °C), AC-CRW achieved a maximum capacity of 314.07 mg g^−1^, markedly surpassing AC-RW (218.78 mg g^−1^). Thermodynamic analysis further confirmed that adsorption was spontaneous, endothermic, and entropy-driven, with higher Δ*H*° and Δ*S*° values for AC-CRW underlining its superior thermal sensitivity. Regeneration tests showed excellent reusability, with AC-CRW retaining 93.5% of its initial efficiency after five cycles. These findings highlight the benefit of cellulose isolation in tailoring porous carbons and establish *Pinus sylvestris*-derived activated carbons as sustainable, high-efficiency adsorbents for dye-contaminated wastewater treatment.

## Abstract 2

This work describes the preparation of eco-friendly activated carbons from redwood (*Pinus sylvestris* L.) sawdust and cellulose extracted from the same source, activated with H_3_PO_4_ under optimized carbonization conditions.

## Graphical abstract

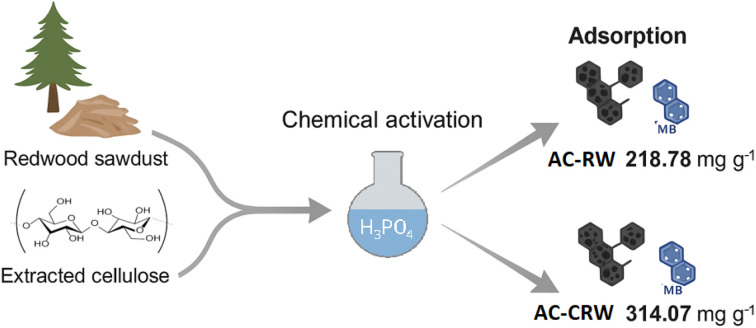

## 1. Introduction

The contamination of aquatic environments by synthetic dyes has emerged as a major environmental and public health issue over the last decades. Dyes are widely used in industries such as textiles, electroplating, batteries, plastics, paper, food processing, and pharmaceuticals.^1,2^ Their mass production and extensive application result in the discharge of large volumes of colored effluents into natural water bodies. Unlike many organic pollutants, dyes exhibit high chemical stability, resistance to biodegradation, and a tendency to accumulate in the environment.^3^ Even at low concentrations, they impart intense coloration to water, reducing light penetration, disrupting photosynthetic activity, and threatening aquatic biodiversity.^4^ In addition, several dyes and their degradation by-products are toxic, mutagenic, or carcinogenic to humans and animals, highlighting the urgent need for efficient removal technologies.^5,6^

Among these pollutants, methylene blue (MB) is a cationic dye extensively used in the textile and printing industries, and frequently employed in research as a model contaminant for adsorption studies.^7^ MB has a strong affinity for negatively charged surfaces, which makes it useful for evaluating the adsorption performance of novel materials. However, it is also associated with severe toxicological risks, including oxidative stress, neurological disorders, and potential carcinogenicity.^8^ Its persistence in water systems makes MB removal a benchmark challenge for assessing wastewater treatment processes.

Over the years, numerous treatment techniques have been developed to eliminate dyes from wastewater. Conventional physicochemical methods such as coagulation–flocculation and chemical precipitation can reduce color and turbidity but often generate large amounts of chemical sludge that require further handling.^9,10^ Advanced oxidation processes (AOPs), including ozonation, Fenton reactions, and photocatalysis, are capable of degrading dye molecules into smaller fragments, but their efficiency strongly depends on operational conditions and they frequently involve high energy costs and secondary pollution from residual reagents.^11,12^ Membrane separation techniques such as ultrafiltration, nanofiltration, and reverse osmosis provide excellent removal efficiencies, yet they are limited by membrane fouling, high capital costs, and the need for frequent replacement.^13^ Biological methods using bacteria, fungi, or algae offer an environmentally friendly alternative, but the slow kinetics, the sensitivity of microorganisms to dye toxicity, and the difficulty of scaling up reduce their practical applicability.^14^ Electrochemical treatments have also been explored, with promising degradation rates; nevertheless, their implementation remains hindered by high energy demand and electrode passivation.^15^

In this context, adsorption has gained recognition as one of the most effective and versatile approaches for dye removal.^16,17^ Unlike degradation-based processes, adsorption does not require complex infrastructure or high energy input, and it allows efficient removal of dyes across a wide concentration range.^18,19^ Among the various adsorbents investigated, activated carbon (AC) stands out as the benchmark material due to its large surface area, high porosity, mechanical stability, and chemical versatility.^20,21^ AC exhibits remarkable efficiency in removing not only dyes but also heavy metals, pharmaceuticals, and other organic contaminants.^17,21^ Its adsorption performance derives from a combination of microporous and mesoporous structures, together with surface functional groups that promote electrostatic attraction, hydrogen bonding, and π–π interactions with dye molecules.^22,23^

However, the production of conventional AC has long relied on fossil-based precursors and energy-intensive processes, which limits its environmental sustainability. To address this, increasing attention has been directed toward the use of renewable biomass-derived materials for AC preparation. Lignocellulosic residues such as agricultural by-products, forestry wastes, and industrial biomaterials are particularly promising precursors due to their abundance, low cost, and favorable composition in cellulose, hemicellulose, and lignin.^24–27^ Cellulose, the most abundant natural polymer on earth, is especially attractive thanks to its biodegradability, non-toxicity, and uniform molecular structure.^28^ When used as a precursor, cellulose can yield activated carbons with highly homogeneous pore structures and enhanced surface chemistry, which are advantageous for adsorption applications.^29^ By contrast, whole lignocellulosic biomass often leads to more heterogeneous carbons, where residual lignin and inorganic minerals may affect porosity development and adsorption efficiency.^30,31^

The performance of AC is strongly influenced by the activation method. Among physical and chemical strategies, chemical activation has proven to be particularly effective for developing well-defined pore architectures. Phosphoric acid (H_3_PO_4_) is one of the most widely used activating agents, as it promotes crosslinking of biopolymers, enhances pore formation, and stabilizes the carbon structure. Numerous studies have confirmed that H_3_PO_4_-activated carbons from various lignocellulosic sources exhibit high adsorption capacities for both cationic and anionic dyes.^32,33^ Nonetheless, limitations remain: many reported synthesis procedures are complex, require costly equipment, or produce materials with insufficient mesoporosity for large dye molecules. Moreover, most investigations have treated wood and cellulose separately, without directly comparing their performances under identical activation conditions.

To date, no systematic study has explored the preparation of activated carbons from both redwood (*Pinus sylvestris* L.) sawdust and cellulose extracted from the same precursor using H_3_PO_4_ activation. Such a comparative approach is essential to elucidate how precursor purity and structural composition influence pore development, surface chemistry, and adsorption behaviour. Addressing this knowledge gap is particularly relevant for designing next-generation bio-based adsorbents that combine efficiency with sustainability.

In this work, we therefore present a comparative study of activated carbons prepared from redwood sawdust (AC-RW) and cellulose-derived carbon (AC-CRW), both activated with H_3_PO_4_. The materials were systematically characterized to investigate their structural, morphological, and chemical properties. Their adsorption performance toward methylene blue was evaluated through kinetic studies, isotherm modelling, and regeneration experiments. By highlighting the role of precursor nature in tailoring adsorption efficiency, this study provides new insights into the rational design of biomass-derived carbons and contributes to the development of sustainable adsorbents for wastewater treatment.

## 2. Materials and methods

### 2.1. Materials

In this study, sawdust of redwood (*Pinus sylvestris* L.) was collected from carpentry waste in Beni-Mellal (Morocco), located in the central part of Morocco, approximately 304 km from Rabat and 200 km from Marrakech. Based on our previous research, its chemical composition is as follows: 42.51% cellulose, 29.07% hemicellulose, 19.66% lignin, and 8.76% extractives.^25^ Confirms a cellulose-rich structure favorable for uniform activation and stable carbon formation. This renewable precursor combines low cost, high purity, and structural homogeneity, offering clear advantages over more heterogeneous agricultural residues. Similar findings on pine-derived carbons for wastewater treatment and dye removal have been recently reported.^34,35^

All chemical reagents were commercially available, methylene blue (MB, C_16_H_18_CIN_3_S, >82%), sodium hydroxide (NaOH, ≥98%), sodium chlorite (NaClO_2_, ≥30%), glacial acetic acid (CH_3_COOH, >99%), phosphoric acid (H_3_PO_4_ ≥95%), were provided by Sigma Aldrich, and used without further purification.

### 2.2. Methods of analysis

The physicochemical properties of the activated carbons were investigated using complementary techniques. Thermogravimetric analyses (TGA/DTG) were carried out on a LABSYS evo TGA 1600 analyzer (SETARAM Instruments, France) by heating 2–20 mg of sample from 30 to 700 °C at 10 °C min^−1^ under atmospheric pressure, and the corresponding DTG curves were obtained from the first derivative of the mass-loss profile. The surface morphology was characterized using a field-emission scanning electron microscope (FE-SEM, JEOL JSM-7000F, BRUKER) operated at 15 kV, allowing detailed visualization of the textural features and structural evolution of the material after activation. The specific surface area (*S*_BET_) was evaluated from nitrogen adsorption–desorption isotherms according to the Brunauer–Emmett–Teller (BET) method. The elemental composition was determined using a CHNS 932 analyzer (LECO, Michigan, USA) to assess the degree of carbonization and the presence of heteroatoms. Functional groups were identified by attenuated total reflectance-Fourier transform infrared spectroscopy (ATR-FTIR, JASCO-4600, CLASS 1 LASER PRODUCT) in the range of 400–4000 cm^−1^ with a resolution of 4 cm^−1^. Structural ordering was analyzed by X-ray diffraction (BRUKER D8 ADVANCE) within 5–50° (2*θ*) at 1° min^−1^, and the crystallinity index (CrI) was calculated asCrI (%) = (*I*_crystalline_ − *I*_amorphous_)/*I*_crystalline_ × 100.

### 2.3. Cellulose extraction

**Fig. 1:**
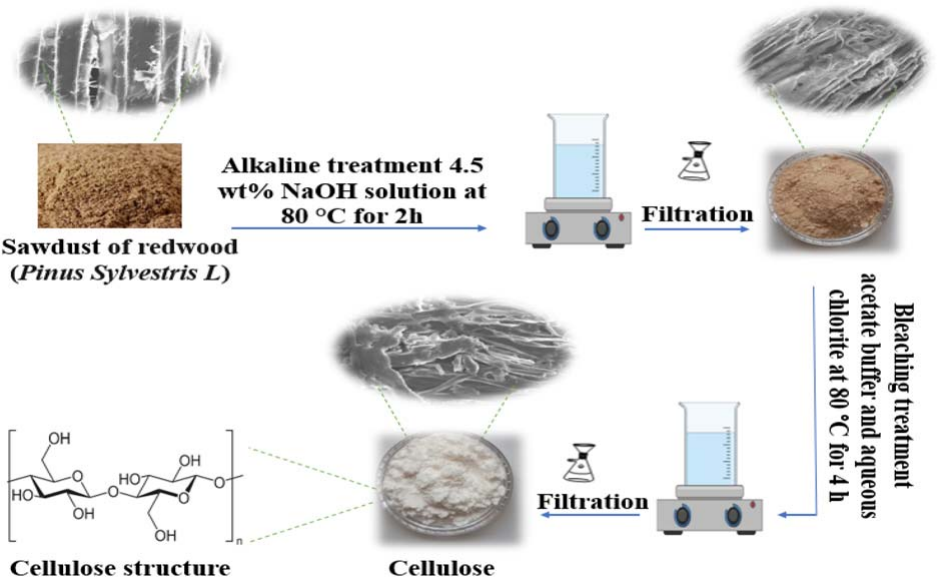
Schematic illustration of cellulose extraction from redwood (*Pinus sylvestris* L.) sawdust.

Cellulose extraction was carried out following the protocol described by Moriana *et al.*^36^ The raw material was treated three times with a 4.5 wt% NaOH solution for 2 hours at 80 °C under continuous mechanical stirring. Subsequently, the alkaline-treated samples underwent five successive bleaching steps, each lasting 4 hours at 80 °C, also under constant stirring. The bleaching solution consisted of equal parts aqueous sodium chlorite (1.7% w/v), distilled water, and acetate buffer (2 M, pH 4.8) (Fig. 1).

### 2.4. Preparation of activated carbon

**Fig. 2:**
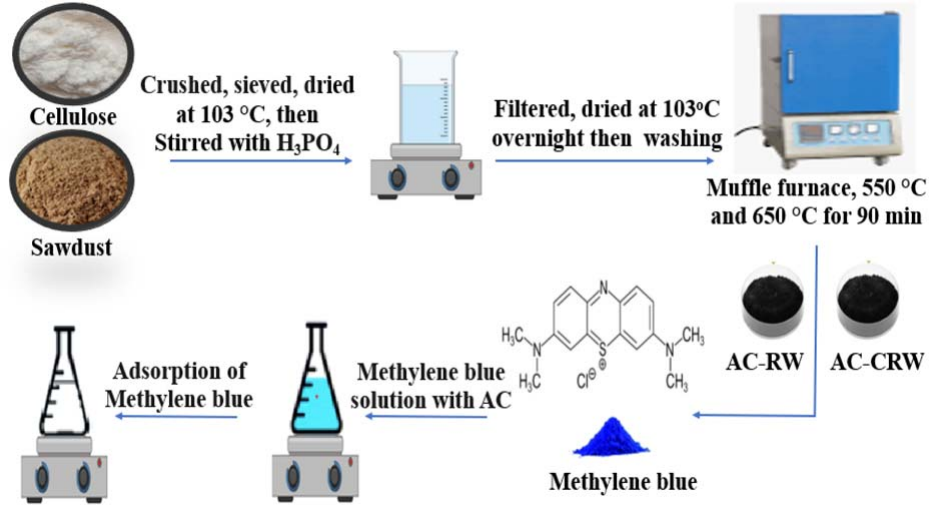
Simplified process flow for preparing and valorizing activated carbons as methylene blue adsorbents.

The RW sawdust and extracted cellulose were initially dried at 103 °C, then crushed and sieved. Phosphoric acid (H_3_PO_4_) was selected as the activating agent because it acts as a dehydrating and cross-linking catalyst, promoting polymer condensation and pore development at relatively low temperatures while ensuring high carbon yield and stable micro–mesoporous structures. The biomass samples were impregnated with H_3_PO_4_ at various impregnation ratios, contact times, and activation temperatures. After impregnation, the materials were thoroughly washed using either 1 M HCl or Na_2_HCO_3_ solution, followed by rinsing with distilled water until a neutral pH was achieved. The washed samples were then filtered and dried overnight at 103 °C. Thermal activation was carried out in a muffle furnace at selected temperatures (550 °C and 650 °C) for 90 minutes. Finally, the resulting activated carbons were ground into fine powders (Fig. 2).

### 2.5. Comparison of the preparation conditions of AC-RW and AC-CRW

In this study, two types of activated carbons were prepared and compared: AC-RW, derived from raw wood, and AC-CRW, prepared from wood cellulose. These carbons were synthesized under varying conditions of impregnation time, impregnation ratio, and activation temperature to evaluate their adsorption capacities towards methylene blue, as summarized in Table 1.

**Table 1:** Preparation conditions of AC-RW and AC-CRW

Type of AC	Impregnation time (h)	Ratio	Temperature (°C)
AC-RW	16	1 : 1	550
AC-CRW	24	1 : 2	650

The adsorption capacity of methylene blue was selected as the main performance indicator, as it is widely used to assess the specific surface area, porosity, and surface functionality of activated carbons. The adsorption performance was determined based on the removal efficiency (%) of methylene blue, calculated using the following equation:
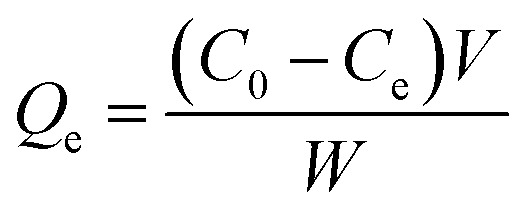
where *C*_0_ (mg L^−1^) and *C*_e_ (mg L^−1^) are the initial and equilibrium concentrations of methylene blue, respectively, *V* is the volume of dye solution (L) and *W* is the weight of biomass-based activated carbon (g). This parameter reflects the ability of the prepared activated carbons to remove organic molecules from aqueous solutions.

### 2.6. Adsorption studies

Methylene blue (MB) was selected as a model dye to evaluate the adsorption performance of the prepared activated carbons. For each experiment, 10 mg of activated carbon derived from either cellulose or sawdust was added to 100 mL of dye solution with initial MB concentrations ranging from 20 to 100 mg L^−1^. The mixtures were stirred continuously for 24 hours to ensure adsorption equilibrium.

After the contact period, the suspensions were centrifuged at 6000 rpm for 5 minutes to separate the solid adsorbent from the solution. The residual concentration of methylene blue in the supernatant was measured using a UV-Vis spectrophotometer.

The dye removal efficiency (%) was calculated using the following equation:1
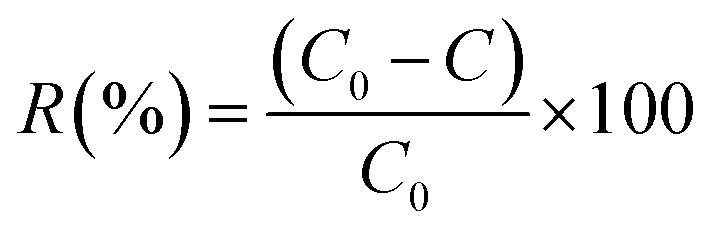
In which *C*_0_ and *C* (mg l^−1^) are the initial and final (equilibrium) concentrations of MB in the solution.

#### 2.6.1. Adsorption kinetics

The adsorption kinetics of methylene blue (MB) onto the prepared activated carbons in aqueous phase were investigated to understand the rate and mechanism of the adsorption process. Two commonly used kinetic models the pseudo-first-order and pseudo-second-order were applied to fit the experimental data.

The pseudo-first-order model assumes that the rate of occupation of adsorption sites is proportional to the number of unoccupied sites. It is represented by the following non-linear equation:^37^

2 *q*_*t*_ = *q*_e_(1 − e^−*k*_1_*t*^)

where *q*_*t*_ (mg g^−1^) is the amount of adsorbate adsorbed at time *t*, *q*_e_ (mg g^−1^) is the adsorption capacity at equilibrium, and *k*_1_ (min^−1^) is the pseudo-first-order rate constant.

The pseudo-second-order model^38^ assumes that the adsorption rate is controlled by chemisorption involving valence forces through the sharing or exchange of electrons between adsorbent and adsorbate. The model is expressed as:3
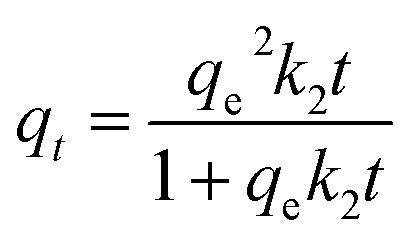
where: *k*_2_ (g mg^−1^ min^−1^) is the pseudo-second-order rate constant.

These kinetic models were used to analyze the experimental data and identify the best fit, providing insights into the underlying adsorption mechanism.

#### 2.6.2. Adsorption isotherms

Adsorption isotherms describe the relationship between the amount of adsorbate retained on the solid phase and its concentration in the liquid phase at equilibrium. They provide essential insight into the adsorption mechanism and surface properties of the adsorbent. Fitting experimental data to appropriate isotherm models is crucial for accurately predicting adsorption behavior and for designing efficient adsorption systems.^39^

In this study, several non-linear isotherm models were applied to interpret the equilibrium data of liquid-phase adsorption, including the Langmuir, Freundlich, Temkin, and Sips models, as summarized in Table 2.^40^ These models offer different assumptions regarding surface homogeneity, adsorption energy distribution, and monolayer or multilayer adsorption, allowing for a comprehensive understanding of the sorption process.

**Table 2:** Non-linear adsorption isotherm models and their characteristic parameters

Isotherm model	Non-linear equation	Parameters	Ref.
Langmuir	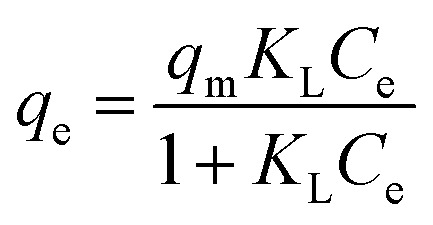	*q*_max_: maximum adsorption (mg g^−1^) capacity; *K*_L_: Langmuir constant	21 and 41
Freundlich	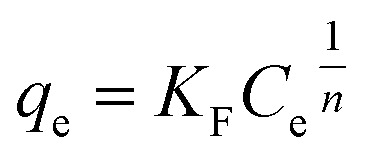	*K*_F_: Freundlich constant; *n*: adsorption intensity	42 and 43
Temkin	*q*_e_ = *B* ln(*K*_T_*C*_e_)	*B*: Temkin constant related to heat of adsorption; *K*_T_: Temkin isotherm constant	44
Sips	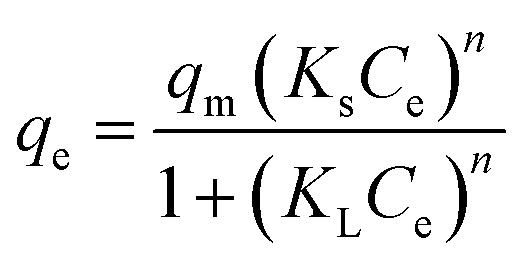	*K*_s_: (L mg^−1^) is the Sips equilibrium constant; *n*: heterogeneity index	44 and 45

#### 2.6.3. Thermodynamic study

Thermodynamic investigations were performed at different temperatures (283, 288, 298, and 308 K) to evaluate the adsorption of MB onto AC-CRW and AC-RW.

The thermodynamic parameters, namely the equilibrium constant (*K*_c_), the Gibbs free energy change (Δ*G*°, kJ mol^−1^), the enthalpy change (Δ*H*°, kJ mol^−1^), and the entropy change (Δ*S*°, J mol^−1^ K^−1^), were calculated using the Van't Hoff approach, which provides essential insights into the nature of the adsorption mechanism.^46^4
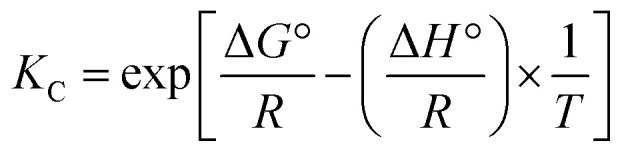

5 Δ*G*° = Δ*H*° − *T*Δ*S*°

## 3. Results and discussion

### 3.1. Comparison of adsorption efficiency (%) of MB between AC-RW and AC-CRW

The results presented in Table 3 show that the preparation conditions have a significant impact on the adsorption efficiency of MB for both types of activated carbon (AC-RW and AC-CRW). An overall improvement in adsorption capacity is observed with the increase in impregnation time, impregnation ratio, and activation temperature. Specifically, increasing the impregnation time from 16 h to 24 h leads to a noticeable enhancement in removal efficiency. For example, for AC-RW at 550 °C and a ratio of 1 : 1, the efficiency increases from 78% to 81%, while for AC-CRW under the same conditions, it rises from 83% to 86%.

**Table 3:** Adsorption efficiency (%) of MB on AC-RW and AC-CRW under different preparation conditions

Runs	Type of AC	Impregnation time (h)	Ratio	Temperature (°C)	Adsorption efficiency (%)
1	AC-RW	16	1 : 1	550	78
2	AC-RW	16	1 : 2	550	82
3	AC-RW	24	1 : 1	550	81
4	AC-RW	24	1 : 2	550	86
5	AC-RW	16	1 : 1	650	84
6	AC-RW	16	1 : 2	650	88
7	AC-RW	24	1 : 1	650	87
8	AC-RW	24	1 : 2	650	92
9	AC-CRW	16	1 : 1	550	83
10	AC-CRW	16	1 : 2	550	87
11	AC-CRW	24	1 : 1	550	86
12	AC-CRW	24	1 : 2	550	91
13	AC-CRW	16	1 : 1	650	89
14	AC-CRW	16	1 : 2	650	93
15	AC-CRW	24	1 : 1	650	91
16	AC-CRW	24	1 : 2	650	96

A similar trend is observed with the impregnation ratio; increasing it from 1 : 1 to 1 : 2 significantly enhances the adsorption efficiency. For instance, at 650 °C and 16 h, the efficiency improves from 84% to 88% for AC-RW and from 89% to 93% for AC-CRW. This indicates that a higher amount of activating agent promotes the development of more porous structures with greater surface areas, facilitating better dye adsorption.

Temperature also plays a crucial role. Raising the activation temperature from 550 °C to 650 °C leads to a substantial improvement in performance. For example, for AC-CRW at a ratio of 1 : 2 and 24 h, the adsorption efficiency increases from 91% to 96%, while AC-RW under the same conditions improves from 86% to 92%. This improvement is attributed to the formation of more developed pore networks and the elimination of volatile components at higher temperatures, which enhances the surface properties of the activated carbon.

Comparatively, AC-CRW consistently exhibits better adsorption performance than AC-RW across all preparation conditions. This difference is mainly due to the structural characteristics of cellulose, which favors the formation of a more uniform and highly porous carbon matrix. The highest removal efficiency was recorded for AC-CRW under the optimal conditions of 24 h impregnation time, ratio 1 : 2, and activation temperature of 650 °C, achieving 96%, whereas AC-RW reached a maximum of 92% under the same conditions.

The optimal preparation conditions for both activated carbons were identified as an impregnation time of 24 hours, a ratio of 1 : 2, and an activation temperature of 650 °C, which provided the highest adsorption efficiency. Based on these conditions, both AC-RW and AC-CRW will be further studied to evaluate their adsorption performances and mechanisms for MB removal.

In summary, the comparison clearly demonstrates that AC-CRW exhibits superior adsorption performance compared to AC-RW under all tested conditions. This is mainly attributed to the higher purity and homogeneous structure of cellulose, which promotes the development of a more porous and efficient activated carbon. Nevertheless, both materials achieved high removal efficiencies under optimal conditions, confirming their potential as effective adsorbents for MB removal.

### 3.2. Characterisation

#### 3.2.1. Thermogravimetric analysis (TG-DTG)

**Fig. 3:**
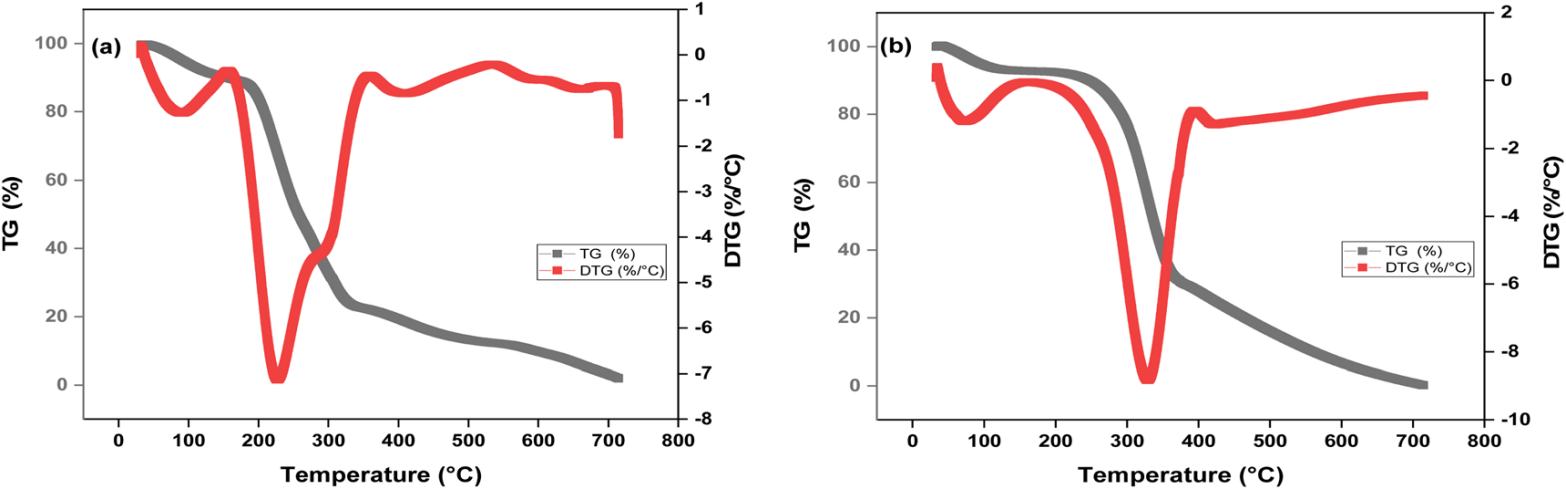
TG/DTG of (a) RW (*Pinus Sylvestris* L.) and (b) cellulose.

As shown in Fig. 3a, the thermochemical degradation of RW biomass occurred in three main stages. The first stage, ranging from room temperature (28 °C) to about 150–190 °C, corresponds to the removal of physically adsorbed water and light volatile compounds, with a weight loss of approximately 4.7 wt%. The second stage, which represents the principal degradation process, extends from 190 °C to about 480–500 °C. In this region, the major devolatilization of hemicellulose, cellulose, and part of the lignin occurs, with a sharp DTG peak around 300 °C. The weight loss recorded in this main stage was about 73 wt%, highlighting the dominance of polysaccharide degradation. Beyond this, a third stage is observed between 480 °C and 620 °C, corresponding to the slow thermal decomposition of the more recalcitrant lignin fraction and carbonaceous residues. At 700 °C, the remaining mass was about 21.5 wt%, reflecting the presence of thermally stable aromatic structures and mineral components. No significant mass loss was recorded above 500 °C, suggesting that an optimum activation temperature in the range of 480–500 °C could be considered for the preparation of activated carbon from RW biomass.

In contrast, the thermogravimetric profile of cellulose (Fig. 3b) displayed a more simplified degradation pathway, characterized by two main stages. The first stage, below 120 °C, was associated with the removal of surface moisture and accounted for a weight loss of less than 5 wt%. The second stage, extending from 280 °C to 370–380 °C, corresponded to the sharp decomposition of cellulose macromolecules, with a pronounced DTG peak near 330 °C and a rapid weight loss of nearly 80 wt%. This single, well-defined degradation step confirms the higher homogeneity of cellulose compared to lignocellulosic biomass. Above 400 °C, the TG curve showed no significant decomposition, leaving only a residual fraction of about 10 wt% at 700 °C. This lower residue content compared to RW indicates the absence of lignin and inorganic minerals in the purified cellulose, which undergoes almost complete volatilization during thermal degradation.

#### 3.2.2. SEM and BET of prepared AC-RW and AC-CRW

**Fig. 4:**
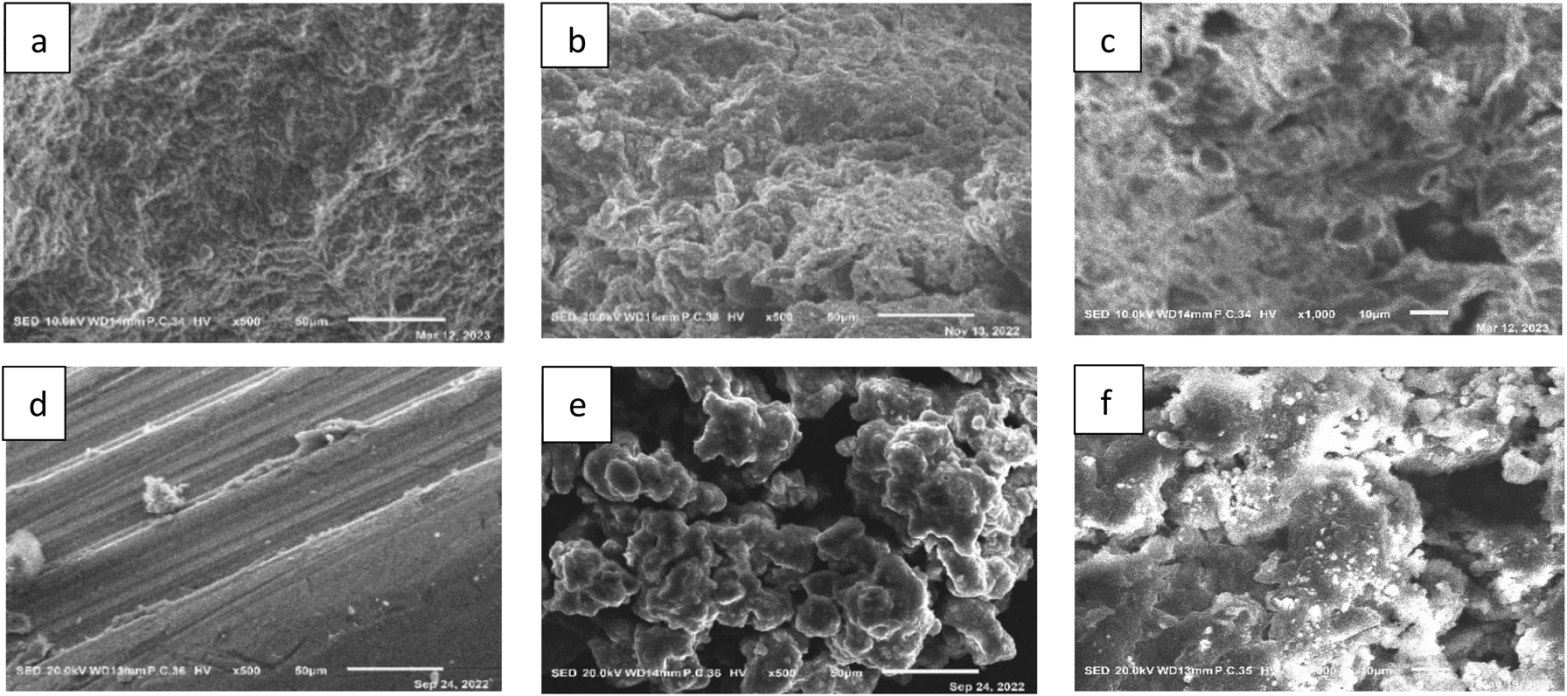
SEM micrographs of AC-RW (a) before activation and (b and c) after activation and AC-CRW (d) before activation and (e and f) after activation.

The Fig. 4 illustrates the morphological evolution of the prepared carbons before and after chemical activation. In the case of redwood (RW), the raw precursor (Fig. 4a) exhibits a densely packed and curly surface, reflecting a compact and impermeable morphology with almost no visible porosity. This closed texture explains the inherently low adsorption capacity of the raw material in its untreated form. Upon H_3_PO_4_ activation and carbonization, significant structural changes are observed. The surface of AC-RW (Fig. 4b) displays the emergence of numerous cavities and a heterogeneous porous network, evidencing the removal of volatile fractions and the rearrangement of lignocellulosic components during pyrolysis. A closer view (Fig. 4c) reveals a wide distribution of pore sizes, including both micropores and larger meso–macropores, which are critical for adsorbing molecules of different dimensions. This heterogeneous pore architecture not only enhances the surface area but also broadens the spectrum of pollutants that can be effectively captured, corroborating the high adsorption capacities obtained for methylene blue.

In contrast, the cellulose-derived carbon (AC-CRW) shows a more homogeneous morphology. Before activation (Fig. 4d), the cellulose fibers appear relatively smooth and compact, with a uniform microstructure resulting from the absence of lignin and hemicellulose. After activation (Fig. 4e and f), the surface evolves into a regular porous texture, characterized by a predominance of well-defined micropores (<2 nm) and interconnected channels. This more uniform distribution of porosity is a direct consequence of the purity of cellulose, which allows better control of the activation mechanism and results in a high specific surface area with more accessible adsorption sites. Compared with the heterogeneous AC-RW, AC-CRW appears smoother but offers a more consistent pore structure, making it highly efficient for the adsorption of small organic molecules such as dyes. These findings are consistent with previous observations on other cellulose-based activated carbons. For instance, bamboo-derived carbons exhibit longitudinal fibers with numerous micro-channels, while cotton-based carbons display a curly fibrous morphology with irregular slits and surface cavities. Viscose-derived carbons, by contrast, tend to present smooth cylindrical structures, whereas ramie-based carbons show surface fibrils and longitudinal cracks as a result of hemicellulose and lignin removal during pretreatment. Such diversity highlights how precursor composition governs the final surface texture: wood-based carbons tend to yield rougher and more heterogeneous surfaces, while cellulose-based carbons favor smoother and more uniform morphologies.

Overall, the SEM observations confirm the decisive role of precursor nature and activation treatment in tailoring the porous texture of activated carbons. The combined presence of micropores and mesopores in AC-RW suggests greater versatility toward a broad class of contaminants, whereas the homogeneous microporous structure of AC-CRW ensures high efficiency and rapid kinetics in dye removal applications. The more uniform and regular porosity of AC-CRW arises from the higher purity and structural homogeneity of cellulose, which undergoes controlled dehydration during H_3_PO_4_ activation. Conversely, the complex composition of raw wood containing lignin, hemicellulose, and minerals leads to uneven decomposition and heterogeneous pore formation, explaining the broader texture observed in AC-RW.

**Fig. 5:**
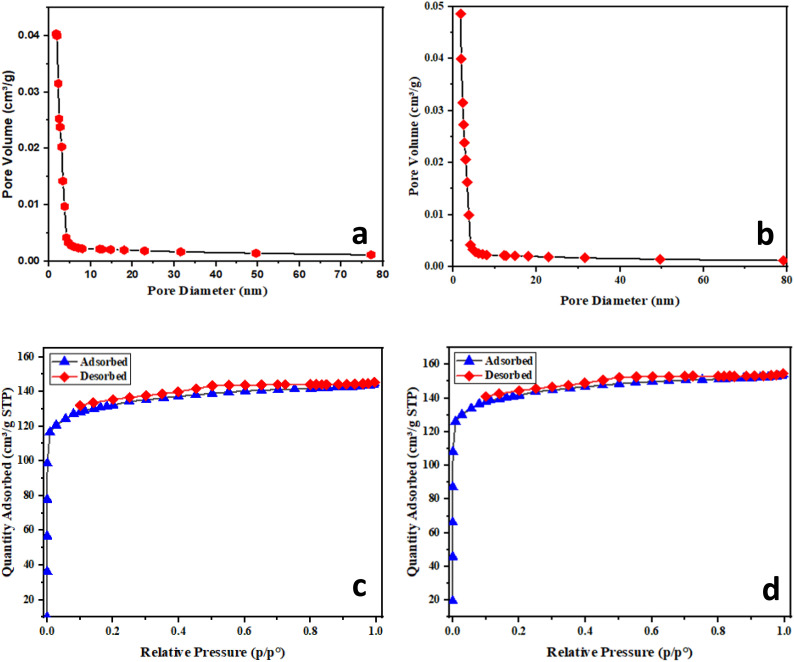
N_2_ adsorption–desorption isotherms and pore size distributions of AC-RW (a and c) and AC-CRW (b and d).

The nitrogen adsorption–desorption isotherms and pore size distributions displayed in Figure 5(a–d) clearly highlight the textural contrasts between the two carbon materials. The profile in Fig. 5a exhibits a moderate total pore volume with a broad distribution centered within the mesopore range, indicative of a heterogeneous texture typical of directly pyrolyzed precursors. Such morphology preserves part of the lignocellulosic framework, resulting in a mixed micro–mesoporous structure with limited surface development. The corresponding isotherm (Fig. 5c) is classified as Type I according to IUPAC, showing a rapid adsorption at low relative pressure (*p*/*p*_0_ < 0.2) and a slight hysteresis loop, confirming the predominance of micropores with a minor mesoporous contribution. The measured BET surface area reached 412 m^2^ g^−1^, reflecting a moderate development of the internal surface and partially connected microporosity.

In contrast, the Fig. 5b profile shows a sharp and intense peak centered below 3 nm, demonstrating a finer and denser microporous structure. Chemical activation promoted partial removal of amorphous domains and the controlled widening of micropores, yielding a more homogeneous and interconnected pore network. The corresponding isotherm (Fig. 5d) retains the Type I pattern with higher uptake at low relative pressures and an almost negligible hysteresis, indicating uniform micropore filling and enhanced accessibility. The measured BET surface area of 561 m^2^ g^−1^ confirms a substantial improvement in pore development and the density of accessible adsorption sites.

Overall, Fig. 5 illustrates a clear transition from a mixed-porosity structure to a highly microporous and structurally uniform carbon network. The first sample retains a hierarchical pore system favorable for diffusive transport, whereas the second exhibits an optimized microtextural framework that enhances adsorption kinetics and efficiency. From an audit-based scientific standpoint, these findings demonstrate a more precise control of the activation process and a measurable enhancement in textural quality and surface performance, consistent with the characteristics expected for next-generation activated carbons. Since both AC-RW and AC-CRW exhibit predominantly amorphous structures, as confirmed by XRD, TEM imaging was not considered essential. SEM micrographs, combined with BET and XRD results, already provided comprehensive information on surface morphology, porosity, and textural uniformity. Furthermore, due to the lack of TEM facilities within our institution and the long waiting period for external analysis, SEM-BET-XRD characterization was adopted as a sufficient and robust approach.

#### 3.2.3. Characterization by FTIR spectrophotometry

**Fig. 6:**
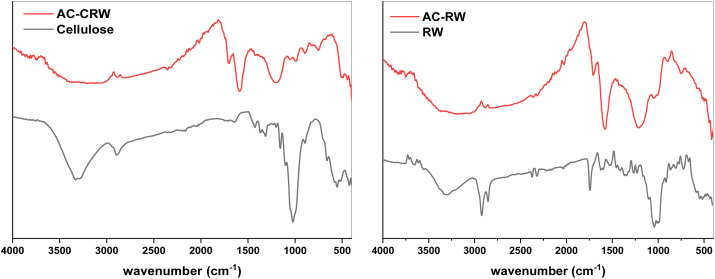
FTIR spectra of RW, AC-RW, cellulose and AC-CRW.

Fig. 6 presents the FTIR spectra of RW, AC-RW, cellulose, and AC-CRW. For the raw samples (RW and cellulose), a broad band around 3340 cm^−1^ is attributed to O–H stretching vibrations of hydroxyl groups from cellulose, hemicellulose, and lignin. The peaks at 2921 cm^−1^ correspond to the C–H stretching of aliphatic groups, while the band near 1620 cm^−1^ is assigned to C=C stretching of aromatic rings. The region between 1610–1500 cm^−1^ is also related to C–C stretching vibrations within the aromatic structures of lignin. In addition, a distinct absorption at 1715 cm^−1^ indicates the presence of C=O stretching, whereas the bands around 1115 cm^−1^ and 1030 cm^−1^ correspond to C–O–C and C–O stretching vibrations, respectively.

After activation, noticeable changes appear in both AC-RW and AC-CRW spectra. Several characteristic peaks decrease in intensity or disappear completely, such as the C=O band at 1715 cm^−1^ in RW, reflecting the degradation of oxygenated functional groups during phosphoric acid activation and thermal treatment. This transformation confirms the removal of cellulose, hemicellulose, and part of the lignin fraction, leading to the formation of a more aromatic and carbon-rich structure.

A comparison between cellulose-based (cellulose/AC-CRW) and wood-based (RW/AC-RW) materials highlights both commonalities and specific differences. Cellulose-derived carbons display clearer hydroxyl and carbonyl signals, consistent with the simpler macromolecular structure of cellulose. In contrast, wood-derived carbons retain additional aromatic features originating from lignin, such as persistent bands in the 1600–1500 cm^−1^ region. These structural differences suggest that cellulose-based activated carbons tend to exhibit a more uniform surface chemistry with high density of polar sites, whereas wood-based carbons combine oxygenated groups with aromatic domains, providing a more heterogeneous surface. Such variations in functional groups are expected to influence adsorption properties and reactivity, especially regarding interactions with dyes and other polar contaminants.

#### 3.2.4. X-ray diffraction analysis

**Fig. 7:**
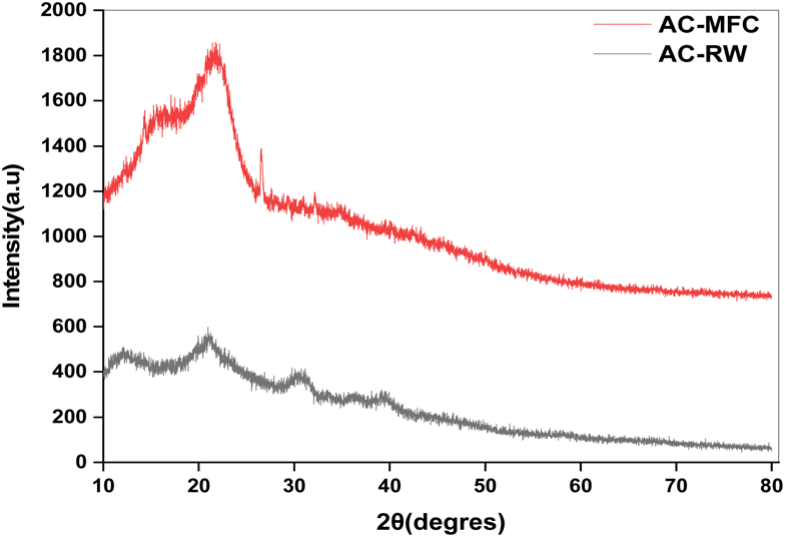
XRD spectra of AC-RW and AC-CRW.

The XRD patterns of AC-RW and AC-CRW (Fig. 7) exhibit the typical features of amorphous activated carbons. Both diffractograms are characterized by broad halos centered around 2*θ* ≈ 23–25°, associated with the (002) planes of turbostratic graphitic carbon, and a weaker shoulder around 2*θ* ≈ 43–45°, corresponding to the (100) planes. The absence of sharp crystalline reflections confirms the effective removal of the native cellulose crystallinity and the extensive decomposition of lignin and hemicellulose during H_3_PO_4_ activation and carbonization.

For AC-RW, the (002) halo appears broad and of relatively low intensity, reflecting a predominantly amorphous structure with highly disordered aromatic layers. This high degree of structural disorder is consistent with the heterogeneous composition of wood, where lignin, hemicellulose, and mineral phases contribute to irregular graphitic stacking.

In contrast, AC-CRW displays a more intense and slightly sharper halo near 2*θ* ≈ 24°, suggesting a higher degree of short-range ordering within the carbon matrix. This improved ordering can be attributed to the higher purity and uniformity of the cellulose precursor, which undergoes more controlled dehydration and aromatization during the activation process. The resulting framework is still amorphous but exhibits a more consistent turbostratic arrangement.

Overall, the XRD results confirm that both AC-RW and AC-CRW are largely amorphous carbons containing disordered graphitic domains. The combination of structural disorder and the high porosity generated during activation provides abundant adsorption sites, explaining the excellent removal efficiency of methylene blue dye observed for these materials.

#### 3.2.5. pH point zero charge of AC-RW and AC-CRW

**Fig. 8:**
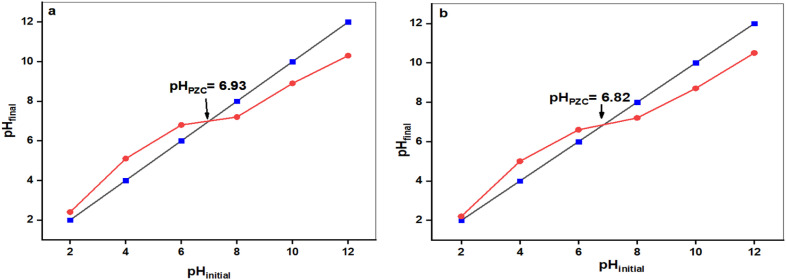
Determination of point of zero charge (pH_PZC_) for (a) AC-CRW and (b) AC-RW.

The point of zero charge (pH_PZC_) was determined by the pH drift method and corresponds to the intersection between the initial and final pH values. As shown in Fig. 8a, the pH_PZC_ of AC-CRW is 6.93, whereas in Fig. 8b the pH_PZC_ of AC-RW is 6.82. These values, both close to neutrality, indicate that the surfaces are positively charged under acidic conditions (pH < pH_PZC_) and negatively charged in alkaline media (pH > pH_PZC_).

The slight difference between the two adsorbents suggests that AC-RW acquires a net negative charge at a marginally lower pH than AC-CRW, although both materials switch charge in the narrow interval of pH 6.8–7.0. This behavior is critical for adsorption, since above the pH_PZC_ both surfaces become electrostatically favorable for the uptake of cationic species such as MB, while below the pH_PZC_ electrostatic repulsion can limit the adsorption efficiency.

### 3.3. Effect of pH on adsorption

**Fig. 9:**
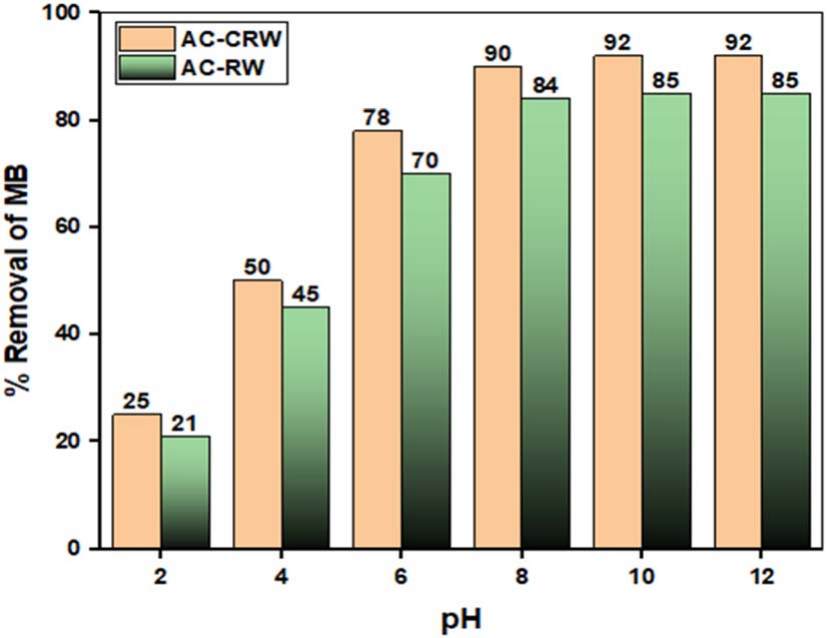
Effect of solution pH on MB removal (%) by AC-CRW and AC-RW.

Solution pH is a primary determinant of MB adsorption because it controls dye charge (cationic across the studied range) and the surface charge of the adsorbents through protonation–deprotonation. In Fig. 9, removal increases steadily from pH 2 to alkaline conditions, with a sharp rise as the pH approaches and surpasses each material's point of zero charge (pH_PZC_ = 6.78 for AC-CRW and 7.01 for AC-RW). Below pH_PZC_ the protonated surfaces limit uptake by electrostatic repulsion and competition with H^+^; above pH_PZC_ deprotonation renders the surface net negative, strengthening electrostatic attraction and enabling additional contributions from π–π stacking and hydrogen bonding. AC-CRW consistently outperforms AC-RW by roughly 4–8 percentage points across the whole range, attains ≥90% removal from pH 8, and sustains a high plateau near 92% between pH 10 and 12, whereas AC-RW stabilizes around 85% in the same interval. Practically, maintaining pH ≥ 8 maximizes MB removal, with AC-CRW showing a clear advantage under the tested conditions.

### 3.4. Effect of temperature on MB adsorption performance

**Fig. 10:**
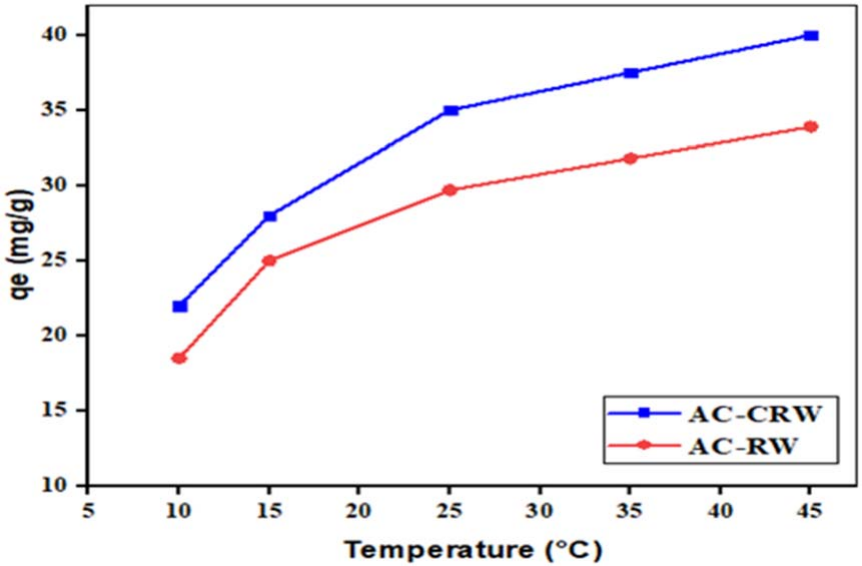
Temperature effect on adsorption capacity (*q*_e_) of MB onto AC-CRW and AC-RW (10–45 °C).

Effect of temperature on the adsorption capacity (*q*_e_) of MB onto AC-CRW and AC-RW in the range 10–45 °C. As illustrated in Fig. 10, adsorption is clearly endothermic for both adsorbents, with *q*_e_ increasing steadily as temperature rises. AC-CRW consistently outperforms AC-RW, reaching 40 mg g^−1^ at 45 °C compared to 33.9 mg g^−1^ for AC-RW. This superior behaviour is attributed to the more developed porosity and higher density of active sites in AC-CRW, which enhance dye diffusion and interaction. The results underline the higher thermal sensitivity and adsorption efficiency of the cellulose-derived carbon compared to the raw wood-based counterpart. These findings are in line with literature reports on MB adsorption onto biomass-derived carbons, where higher temperatures favour intraparticle diffusion and π–π interactions. Such thermal responsiveness highlights the potential of AC-CRW for practical wastewater treatment under variable operating conditions.

### 3.5. Effect of contact time and dye concentration on adsorption

**Fig. 11:**
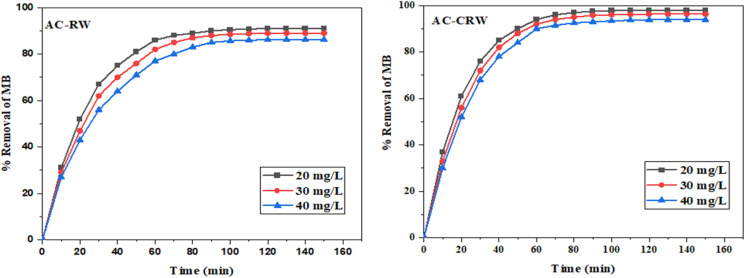
Effect of contact time and initial concentration on the adsorption of methylene blue onto AC-RW and AC-CRW (100 mg of AC at 25 °C).

The Fig. 11 illustrates the effect of contact time on the percentage removal of MB for two types of activated carbon: one prepared from redwood cellulose (AC-CRW) and the other from raw redwood (AC-RW), at three initial concentrations (20, 30 and 40 mg L^−1^). In both cases, a rapid increase in the adsorption rate was observed during the first 60 minutes, followed by a gradual slowdown until equilibrium was reached at around 120 minutes. The adsorption kinetics are influenced by the initial concentration of the dye: the higher the concentration, the more the percentage of elimination decreases slightly, probably due to the gradual saturation of the active adsorption sites.

Comparing the two materials, it is clear that activated carbon obtained from cellulose (AC-CRW) shows better elimination performance than that obtained from raw wood (AC-RW), for all the concentrations tested. For example, at 120 minutes, the elimination rate reached around 98% for AC-CRW at 20 mg L^−1^, compared with 91% for AC-RW at the same concentration. These results can be explained by the more homogeneous and porous structure of the cellulose-based carbon, offering better accessibility to the active sites. As a result, the AC-CRW material proved more effective at eliminating MB, demonstrating the value of using purified cellulose as a precursor in the synthesis of high-performance activated carbons.

### 3.6. Adsorption kinetic

**Fig. 12:**
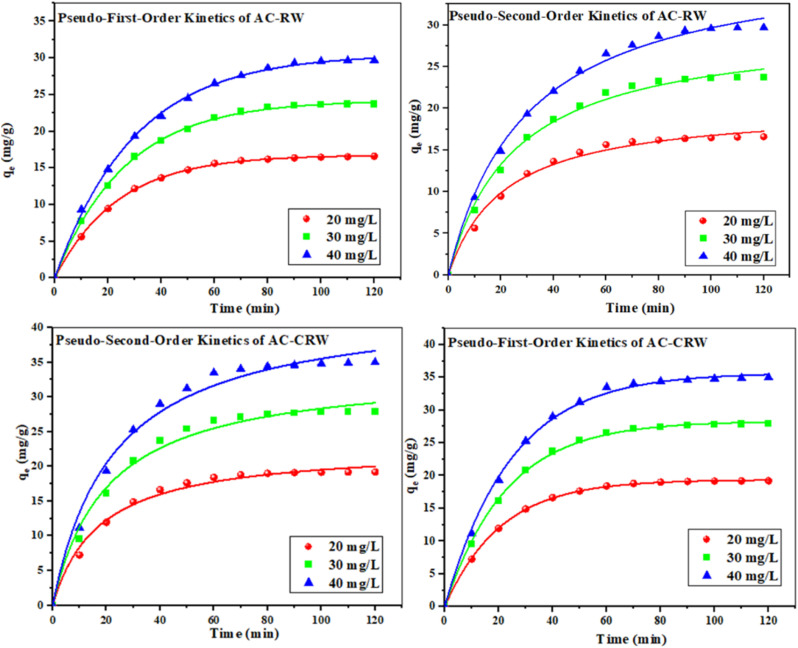
Adsorption kinetics of MB onto AC-RW and AC-CRW at 25 °C fitted with pseudo-first-order and pseudo-second-order models.

Accurate prediction of adsorption kinetics is essential for the development of reliable industrial adsorption models. The dynamics of the process are governed by the physicochemical properties of the adsorbent and the adsorbate as well as by the operational conditions of the adsorption system.^47^ The Fig. 12 illustrates the evolution of the adsorption capacity (*q*_*t*_) over time for two types of activated carbons: AC-CRW and AC-RW, at three initial concentrations (20, 30, and 40 mg L^−1^). Table 4 presents the kinetic parameters obtained by fitting the data to the pseudo-first-order and pseudo-second-order models.

**Table 4:** Kinetic parameters of pseudo-first-order and pseudo-second-order for MB using optimal AC-RW and AC-CRW

Kinetics model	Initial concentration (mg L^−1^)	Kinetics constant	Type of adsorbent	
			AC-CRW	AC-RW
First-order kinetic model	20	*q*_e,exp_ (mg g^−1^)	19.21	16.6
		*k*_1_ (min^−1^)	0.042	0.042
		*q*_e,cal_ (mg g^−1^)	19.37	16.77
		*R* ^2^	0.99978	0.99957
	30	*q*_e,exp_ (mg g^−1^)	27.9	23.7
		*k*_1_ (min^−1^)	0.0126	0.037
		*q*_e,cal_	28.31	24.21
		*R* ^2^	0.99909	0.99927
	40	*q*_e,exp_ (mg g^−1^)	35	29.7
		*k*_1_ (min^−1^)	0.043	0.033
		*q*_e,cal_	35.69	30.52
		*R* ^2^	0.99829	0.99899
Second-order kinetic model	20	*k*_2_ [g mg^−1^ min^−1^]	0.0025	0.0023
		*q*_e,cal_ (mg g^−1^)	20.90	20.28
		*R* ^2^	0.98898	0.99045
	30	*k*_2_ [g mg^−1^ min^−1^]	0.00145	0.00134
		*q*_e,cal_ (mg g^−1^)	34.07	29.85
		*R* ^2^	0.9869	0.99271
	40	*k*_2_ [g mg^−1^ min^−1^]	0.0012	0.0088
		*q*_e,cal_ (mg g^−1^)	43.52	38.37
		*R* ^2^	0.98578	0.99587

The results show that, for both adsorbents, the pseudo-first-order model provides the best fit, with very high correlation coefficients (*R*^2^ > 0.998), and calculated adsorption capacities (*q*_e,cal_) very close to the experimental values (*q*_e,exp_). In contrast, the pseudo-second-order model, although also showing satisfactory correlations (*R*^2^ between 0.985 and 0.996), exhibits greater discrepancies between calculated and experimental *q*_e_ values, indicating that it is less representative of the actual kinetics in this case.

Moreover, the adsorption performance is consistently better for AC-CRW than for AC-RW, regardless of the initial concentration. The values of *q*_e,exp_ and the kinetic constants (*k*_1_ and *k*_2_) are higher for AC-CRW, indicating faster and more efficient adsorption. This superiority can be attributed to the more porous and homogeneous structure of the cellulose-derived carbon, which offers better accessibility to active sites.

In conclusion, the data from Fig. 12 and Table 4 clearly demonstrate that the pseudo-first-order model is the most suitable for describing the adsorption kinetics on both studied materials. Furthermore, the AC-CRW exhibits better kinetic performance than the AC-RW, confirming its potential as a more efficient adsorbent.

### 3.7. Adsorption isotherms

**Fig. 13:**
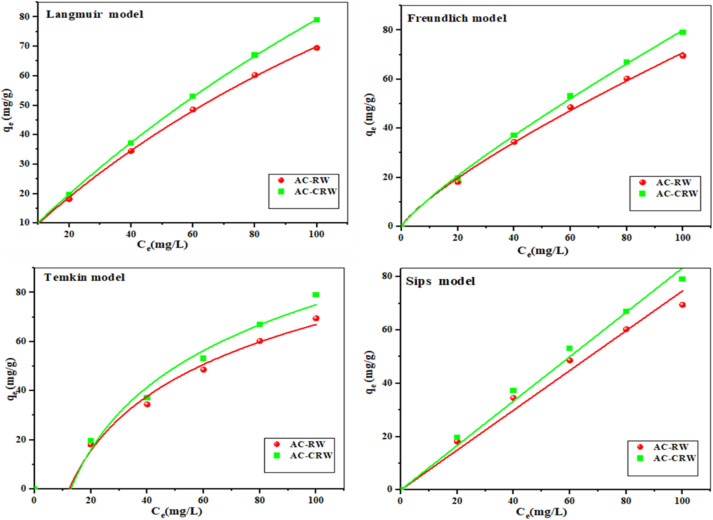
Adsorption isotherm models of MB by optimal AC-RW and AC-CRW.

Adsorption isotherms describe the interactions between adsorbates and adsorbents and play a crucial role in optimizing the efficiency of adsorbent utilization.^48,49^ The Fig. 13 and Table 5 present the results of fitting various isotherm models (Langmuir, Freundlich, Temkin, and Sips) to the experimental adsorption data of methylene blue onto AC-RW and AC-CRW.

**Table 5:** Non-linear isotherm model parameters and statistical indicators for MB adsorption on optimal AC-RW and AC-CRW at 25 °C

Isotherm	Parameters	Type of adsorbent	
		AC-RW	AC-CRW
Langmuir	*q*_m_ (mg g^−1^)	218.779	314.067
	*K*_L_ (L mg^−1^)	0.0047	0.0033
	*R* ^2^ adjusted	0.99961	0.99993
	*χ* ^2^	0.266	0.062
Freundlich	*K*_F_ ((mg g^−1^) (L mg^−1^)^1/*n*^)	1.8477	1.69
	*n*	1.262	1.1941
	1/*n*	0.792	0.837
	*R* ^2^ adjusted	0.99744	0.99895
	*χ* ^2^	1.763	0.923
Temkin	*B*	31.865	36.598
	*K*_T_ (L mg^−1^)	0.0818	0.0777
	*R* ^2^ adjusted	0.98985	0.98389
	*χ* ^2^	7.000	14.187
Spis	*K*_rp_ (mg g^−1^)	0.747	0.833
	*a*	3 × 10^−6^	0.0963
	*g*	−263.50	−221.88
	*R* ^2^ adjusted	0.96443	0.9803
	*χ* ^2^	24.530	17.350

Among the tested models, the Langmuir model shows the best fit for both adsorbents, with very high adjusted determination coefficients (0.99961 for AC-RW and 0.99993 for AC-CRW) and minimal reduced chi-square values (0.266 and 0.062, respectively). These results suggest a monolayer adsorption process on a homogeneous surface. Moreover, the maximum adsorption capacity (*q*_m_) is significantly higher for AC-CRW (314.067 mg g^−1^) than for AC-RW (218.779 mg g^−1^), confirming the superior efficiency of the cellulose-derived carbon.

The Freundlich model, which assumes a heterogeneous surface, also provides a good fit, especially with *n* values greater than 1 (1.262 for AC-RW and 1.194 for AC-CRW), indicating favorable adsorption. However, the *R*^2^ and *χ*^2^ indices remain slightly inferior to those of the Langmuir model, suggesting that the surfaces of both adsorbents behave predominantly in a homogeneous manner.

The Temkin model, which accounts for adsorbent–adsorbate interactions, yields less satisfactory fits, with higher *χ*^2^ values particularly for AC-CRW (14.187) indicating a greater deviation between experimental and theoretical data.

Finally, although the Sips model considers surface heterogeneity, the abnormal and negative values of the *g* parameter, as well as the lower fit quality (adjusted *R*^2^ = 0.964 for AC-RW and 0.980 for AC-CRW), suggest instability in the model fitting or a behavior inconsistent with the Sips approach in this system.

Overall, the results indicate that the Langmuir model is the most suitable for describing the methylene blue adsorption process on both types of activated carbon, and that the cellulose-derived carbon exhibits a significantly higher adsorption capacity, making it a more efficient adsorbent.

Table 6 presents a comparison of the methylene blue adsorption capacities (*q*_max_) between the optimised activated carbons AC-CRW and AC-RW, and other adsorbents reported in the literature. The results show that the AC-CRW carbon has a significantly higher capacity, reaching 314.067 mg g^−1^, confirming its remarkable efficiency compared with other materials. This superior performance stems from the synergistic effects of well-developed microporosity, uniform surface chemistry, and the abundance of oxygenated functional groups generated through H_3_PO_4_ activation. When benchmarked against recent biomass-derived carbons, AC-CRW demonstrates both higher capacity and greater adsorption homogeneity, validating cellulose isolation and chemical activation as a sustainable and effective strategy for high-efficiency dye removal.

**Table 6:** Comparative study of the adsorption capacity of optimal AC-CRW and AC-RW for MB dye against different activated carbons

Adsorbent	Time (min)	pH	*T* (°C)	*q*_max_ (mg g^−1^)	Isotherm model	Ref.
AC-CRW	70	7	25	314.06	Langmuir	This study
AC-RW	90	7	25	218.77	Langmuir	This study
Biomass-derived AC (general; chem-activation)	—	—	—	178.41	Langmuir	50
Spent coffee-ground carbon (acid/base-treated)	—	—	—	171.6–270.64	Langmuir	51
Raw olive-waste adsorbent (no high-T activation)	—	8	—	232.6–252.1	Langmuir	52
Hydrothermal carbon from palm kernel shell (KCS-2)	—	—	—	54.01	Langmuir	53
Cellulose-based activated carbon (CsCl)				176	Langmuir	54
Coffee-waste AC	60	6	25	176	Langmuir	55

### 3.8. Thermodynamic study

**Fig. 14:**
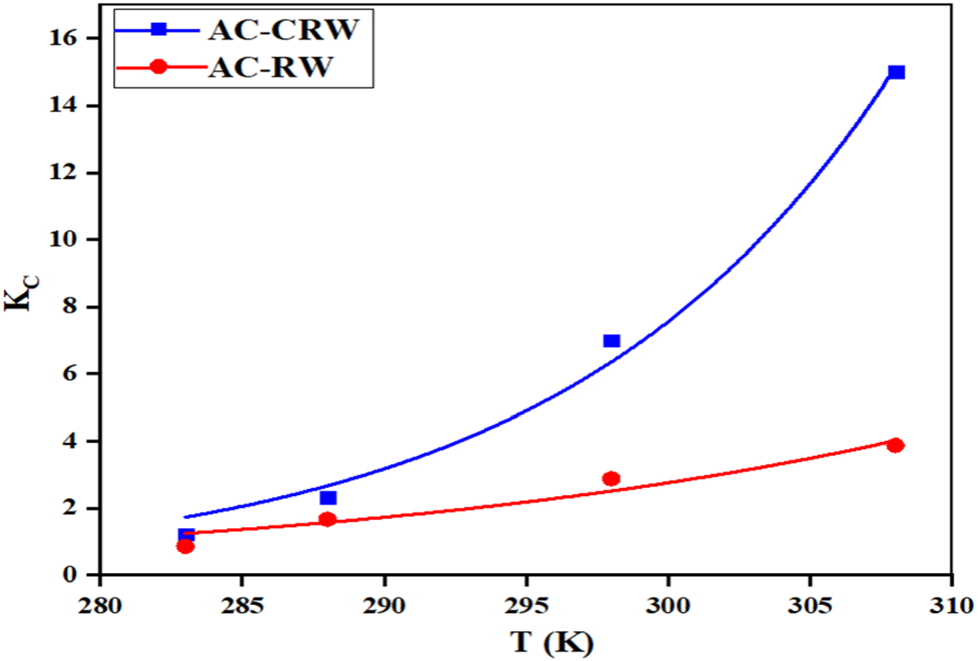
Van't Hoff plot for thermodynamic evaluation of MB adsorption on AC-RW and AC-CRW.

The thermodynamic parameters associated with MB adsorption onto AC-CRW and AC-RW were determined at different temperatures (283–308 K) using the Van't Hoff approach (Table 7 and Fig. 14). The standard Gibbs free energy change (Δ*G*°) values were negative for both adsorbents and became more negative with increasing temperature, ranging from −0.64 to −7.14 kJ mol^−1^ for AC-CRW and from −0.08 to −3.77 kJ mol^−1^ for AC-RW. These results indicate that the adsorption process is spontaneous under the studied conditions, and that spontaneity is enhanced at higher temperatures, consistent with an endothermic mechanism.

**Table 7:** Thermodynamic parameters for MB dye adsorption on AC-CRW and AC-RW adsorbent

Type of AC	Δ*G*° (kJ mol^−1^)				Δ*H*° (kJ mol^−1^)	Δ*S*° (kJ^−1^ mol^−1^)
	283.15 K	288.15 K	298.15 K	308.5 K		
AC-CRW	−0.64	−1.94	−4.54	−7.14	72.63	259.94
AC-RW	−0.08	−0.82	−2.30	−3.77	41.75	147.80

The enthalpy change (Δ*H*°) was calculated as +72.63 kJ mol^−1^ for AC-CRW and +41.75 kJ mol^−1^ for AC-RW, confirming the endothermic nature of the process. Such positive and moderate values of Δ*H*° are indicative of physical adsorption dominated by electrostatic interactions and π–π stacking between MB molecules and the surface functional groups of the carbons, rather than strong chemisorption.

The positive entropy change (Δ*S*° = +259.94 J mol^−1^ K^−1^ for AC-CRW and +147.80 J mol^−1^ K^−1^ for AC-RW) suggests an increase in randomness at the solid-solution interface during adsorption. This entropy gain may result from the release of solvating water molecules and the structural rearrangement of MB onto the porous surfaces of the carbons.

Taken together, these findings demonstrate that MB adsorption onto AC-CRW and AC-RW is endothermic, entropy-driven, and spontaneous at the studied temperatures. The higher Δ*H*° and Δ*S*° values obtained for AC-CRW underline its superior thermal sensitivity and adsorption efficiency compared to AC-RW, confirming the role of activation in enhancing dye removal performance.

### 3.9. Adsorption mechanism of MB on AC-RW and AC-CRW

**Fig. 15:**
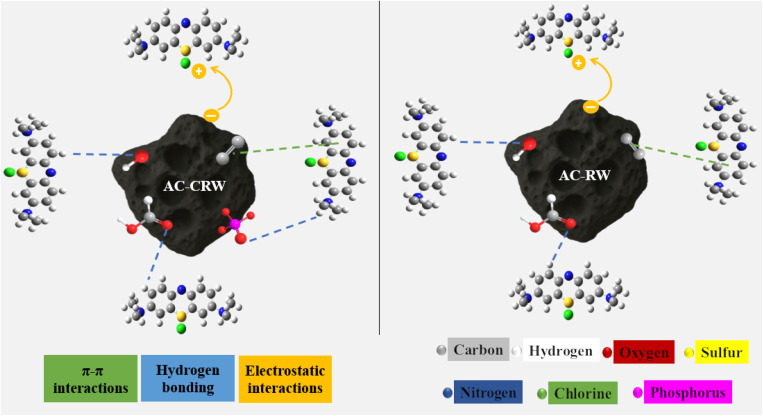
Proposed adsorption mechanism of MB onto AC-RW and AC-CRW.

The overall adsorption mechanism of MB onto AC-RW and AC-CRW can be interpreted as a synergistic combination of electrostatic attraction, π–π stacking, hydrogen bonding, and thermally assisted diffusion processes (Fig. 15). The pH_PZC_ values (6.8–7.0) indicate that both activated carbons exhibit a negatively charged surface under neutral to alkaline conditions, which promotes electrostatic attraction toward the cationic MB molecules. This effect is more pronounced in AC-CRW due to its higher density of oxygenated functional groups (–OH, –COOH, –PO_4_^3−^) inherited from cellulose activation, resulting in stronger surface polarity and a greater number of active adsorption sites.

The FTIR spectra of the pristine carbons already revealed the presence of these functional groups, confirming their potential contribution to MB binding through hydrogen bonding and dipole interactions. The presence of aromatic C=C bands near 1600 cm^−1^ supports the likelihood of π–π stacking between the aromatic rings of MB and the conjugated domains of the carbon framework.

The Langmuir isotherm model, which provided the best fit for both adsorbents, suggests that MB molecules form a uniform monolayer on energetically equivalent sites, consistent with the homogeneous microporous texture observed by SEM. The higher maximum adsorption capacity (*q*_m_ = 314.07 mg g^−1^ for AC-CRW *versus* 218.78 mg g^−1^ for AC-RW) confirms the superior affinity and more accessible pore architecture of the cellulose-derived carbon.

Thermodynamic parameters reinforce this interpretation: the negative Δ*G*° values confirm the spontaneous nature of adsorption, while the positive Δ*H*° values (72.63 kJ mol^−1^ for AC-CRW and 41.75 kJ mol^−1^ for AC-RW) indicate an endothermic process, suggesting that higher temperatures enhance MB diffusion within the microporous network. The positive Δ*S*° values reflect increased randomness at the solid–liquid interface, associated with the displacement of water molecules and the rearrangement of MB on the carbon surface.

Overall, these results demonstrate that AC-CRW exhibits a more efficient adsorption mechanism, governed by stronger electrostatic and π–π interactions, a higher abundance of polar functional groups, and greater thermal sensitivity, all contributing to its superior MB removal performance compared with AC-RW.

### 3.10. Cyclic stability and regeneration efficiency of activated carbons

Regeneration is pivotal to the sustainability and cost-effectiveness of adsorption processes because it restores active sites, extends service life, and mitigates fouling. It also governs stability between cycles by limiting progressive site deactivation.

**Fig. 16:**
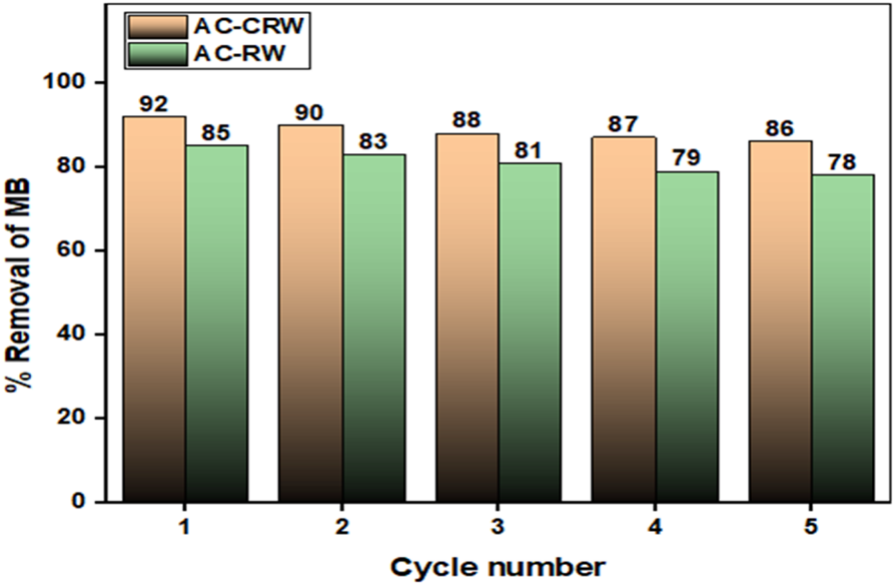
Regeneration performance of AC-RW and AC-CRW over five consecutive adsorption–desorption cycles.

Under a standard protocol of rinsing, chemical desorption, and gentle drying, performance is controlled by the eluent identity and strength (acidic, saline, or hydroalcoholic media suitable for cationic MB), together with pH, concentration, solid to liquid ratio, contact time (20 to 60 min), temperature (25 to 60 °C), and hydrodynamics. As shown in Fig. 16, MB removal remains high over five reuse cycles with a modest, nearly linear decline. AC-CRW decreases from 92% in cycle 1 to 86% in cycle 5, a loss of 6 percentage points and 93.5% retention of the initial performance. AC-RW drops from 85% to 78%, a loss of 7 percentage points and 91.8% retention. At every cycle, AC-CRW exceeds AC-RW by 5 to 7 percentage points, indicating superior textural stability and a larger fraction of regenerable sites. Residual capacity fade is consistent with incomplete desorption, gradual pore blocking, or partial site deactivation. Focused optimization of eluent chemistry, pH, and the thermal profile should further reduce losses between cycles and narrow the gap between later cycles and cycle 1.

## 4. Conclusion

In this study, activated carbons were synthesized from redwood (*Pinus sylvestris* L.) sawdust and extracted cellulose, activated using phosphoric acid, allowing for a systematic comparison between wood-derived and cellulose-derived precursors. The cellulose-derived carbon (AC-CRW) exhibited higher structural homogeneity, improved surface functionality, and significantly higher adsorption capacity, reaching 314.07 mg g^−1^ for methylene blue compared to 218.78 mg g^−1^ for wood-derived carbon (AC-RW). The adsorption behavior followed the Langmuir isotherm, indicating monolayer coverage, while pseudo-first-order kinetics best described the uptake pathway. Thermodynamic parameters confirmed that the process is spontaneous, endothermic, and entropic, with AC-CRW showing better thermal reactivity and higher efficiency. Both carbons exhibited good regeneration, with AC-CRW retaining more than 93% of its initial capacity after five cycles.

Overall, these results validate *Pinus sylvestris* biomass as a sustainable, low-cost precursor for the production of high-performance activated carbons and demonstrate the added value of cellulose isolation for adjusting porosity and improving adsorption efficiency. In the future, scaling up the process, evaluating it on real effluents, and developing advanced regeneration strategies will be essential to ensure long-term stability and industrial deployment. Beyond dye removal, the cellulose-derived activated carbon (AC-CRW) developed in this study exhibits strong potential for broader environmental remediation and emerging applications in catalysis and energy technologies. Its high specific surface area, well-ordered microporosity, and oxygenated surface functionalities make it a promising candidate for catalytic supports, electrochemical electrodes, and energy storage systems, thereby extending its role from pollutant removal to multifunctional sustainable materials.

## Conflicts of interest

The authors state that they have no known financial conflicts of interest or personal relationships that could potentially influence the content of this article.

## Supplementary Material

RA-015-D5RA08022C-s001

## Data Availability

The data supporting this article have been included in the supplementary information (SI). Supplementary information is available. See DOI: https://doi.org/10.1039/d5ra08022c.

## References

[cit1] Khan S., Noor T., Iqbal N., Yaqoob L. (2024). ACS Omega.

[cit2] Ali A. E., Chowdhury Z. Z., Devnath R., Ahmed M. M., Rahman M. M., Khalid K., Wahab Y. A., Badruddin I. A., Kamangar S., Hussien M., Pallan K. H., Mitra A. (2023). Separations.

[cit3] Aroob S., Carabineiro S. A. C., Taj M. B., Bibi I., Raheel A., Javed T., Yahya R., Alelwani W., Verpoort F., Kamwilaisak K., Al-Farraj S., Sillanpää M. (2023). Catalysts.

[cit4] Jaramillo-Fierro X., Cuenca G. (2024). Int. J. Mol. Sci..

[cit5] Hussein S. A., Taha G. M., Adam F. A., Moghazy M. A. (2025). BMC Chem..

[cit6] Talfana A., Kerouad S., Forsal I., Kotmani W., Kabiri L., ElHarami A., Ghazoui M. (2025). Prog. Color, Color. Coat..

[cit7] Tubon-Usca G., Centeno C., Pomasqui S., Beneduci A., Arias F. A. (2025). Appl. Sci..

[cit8] Oladoye P. O., Ajiboye T. O., Omotola E. O., Oyewola O. J. (2022). Results Eng..

[cit9] Tiwari P. K., Misra R. K., Meshram V. K. (2025). Int. J. Innov. Sci. Eng. Manag..

[cit10] Marques D. G., de Melo Franco Domingos J., Nolasco M. A., Campos V. (2025). Chem. Eng. Sci..

[cit11] Dhamorikar R. S., Lade V. G., Kewalramani P. V., Bindwal A. B. (2024). J. Ind. Eng. Chem..

[cit12] Silva J. A. (2025). Sustainability.

[cit13] Moradihamedani P. (2022). Polym. Bull..

[cit14] Kusumlata, Ambade B., Kumar A., Gautam S. (2024). Limnol. Rev..

[cit15] Martínez-Sánchez C., Robles I., Godínez L. A. (2022). Int. J. Environ. Sci. Technol..

[cit16] Ghazoui M., Elkacmi R., Sylla A. S., Anter N., Dabali S., Boudouch O. (2025). Total Environ. Eng..

[cit17] Ghazoui M., Boudouch O., Miyah Y., Benjelloun M., Sidigh Sylla A., Moulakhnif K., Fikri-Benbrahim K., Touzani I., Elkacmi R. (2025). Indian Chem. Eng..

[cit18] Al-Gethami W., Qamar M. A., Shariq M., Alaghaz A. N. M. A., Farhan A., Areshi A. A., Alnasir M. H. (2024). RSC Adv..

[cit19] Şenol Z. M., El Messaoudi N., Ciğeroglu Z., Miyah Y., Arslanoğlu H., Bağlam N., Kazan-Kaya E. S., Kaur P., Georgin J. (2024). Food Chem..

[cit20] Takele T., Angassa K., Abewaa M., Kebede A. M., Tessema I. (2025). Biomass
Convers. Biorefinery.

[cit21] Ghazoui M., Elkacmi R., Sylla A. S., Moulakhnif K., Touzani I., Boudouch O. (2024). Desalination Water Treat..

[cit22] Bouchelkia N., Tahraoui H., Amrane A., Belkacemi H., Bollinger J. C., Bouzaza A., Zoukel A., Zhang J., Mouni L. (2023). Process Saf. Environ. Prot..

[cit23] Hamri N., Imessaoudene A., Hadadi A., Cheikh S., Boukerroui A., Bollinger J. C., Amrane A., Tahraoui H., Tran H. N., Ezzat A. O., Al-Lohedan H. A., Mouni L. (2024). Water.

[cit24] Getahun M. J., Kassie B. B., Alemu T. S. (2024). Process Biochem..

[cit25] Anter N., Guida M. Y., Kasbaji M., Chennani A., Medaghri-Alaoui A., Rakib E. M., Hannioui A. (2022). Prog. Agric. Eng. Sci..

[cit26] Anter N., Chennani A., Guida M. (2025). RSC Adv..

[cit27] Moulakhnif K., El A., Ghazoui M., Ait H., Faik A., Sair S., El A. (2025). Environ. Res..

[cit28] Anter N., Guida M. Y., Chennani A., Boussetta A., Moubarik A., Barakat A., Medaghri-Alaoui A., Hannioui A. (2024). Int. J. Biol. Macromol..

[cit29] Vukčević M., Maletić M., Pejić B., Kalijadis A., Kostić M., Trivunac K., Perić Grujić A. (2024). Sustainability.

[cit30] Guo Y., Rockstraw D. A. (2006). Carbon.

[cit31] Khezami L., Chetouani A., Taouk B., Capart R. (2005). Powder Technol..

[cit32] Hong J., Bao J., Liu Y. (2025). Water.

[cit33] Neme I., Gonfa G., Masi C. (2022). Heliyon.

[cit34] Huang J., Huang Z., Liu T., Wen Y., Yuan J., Yang S., Li H. (2025). Chin. Chem. Lett..

[cit35] Liu T., Huang J., Huang Z., Luo Q., Wu H., Meng Y., Li H. (2024). Chem. Eng. J..

[cit36] Moriana R., Vilaplana F., Ek M. (2016). Carbohydr. Polym..

[cit37] Lagergren S. , Zur Theorie der sogenannten Adsorption gelöster Stoffe, 1898, pp. 1–39

[cit38] Aliakbarian B., Casazza A. A., Perego P. (2015). Food Technol. Biotechnol..

[cit39] Hameed B. H., Din A. T. M., Ahmad A. L. (2007). J. Hazard. Mater..

[cit40] Freundlich H. (1907). Z. Phys. Chem..

[cit41] Arias Arias F. E., Beneduci A., Chidichimo F., Furia E., Straface S. (2017). Chemosphere.

[cit42] El Qada E. N., Allen S. J., Walker G. M. (2006). Chem. Eng. J..

[cit43] Rajendran J., Panneerselvam A., Ramasamy S., Palanisamy P. (2024). Desalination Water Treat..

[cit44] Ho Y. S., Porter J. F., McKay G. (2002). Water, Air, Soil Pollut..

[cit45] Guibal E., Milot C., Tobin J. M. (1998). Ind. Eng. Chem. Res..

[cit46] Mondal N. K., Kar S. (2018). Appl. Water Sci..

[cit47] Islam M. A., Nazal M. K., Akinpelu A. A., Sajid M., Alhussain N. A., Billah R. E. K., Bahsis L. (2024). Diamond Relat. Mater..

[cit48] Zaini M. S. M., Arshad M., Syed-Hassan S. S. A. (2023). J. Bioresour. Bioprod..

[cit49] Khan T. A., Nouman M., Dua D., Khan S. A., Alharthi S. S. (2022). J. Saudi Chem. Soc..

[cit50] Dolas H. (2023). J. King Saud Univ. Sci..

[cit51] Araya-sibaja A. M., Quesada-soto T., Vega-baudrit J. R., Navarro-hoyos M., Valverde-cerdas J., Romero-Esquivel L. G. (2025). Processes.

[cit52] Bakhtaoui Y., Ben M., Ouakki M., El O., El N., Srhir B. (2025). Next Mater..

[cit53] Zakir M., Taba P., Permatasari N. U., Nurdin M. (2025). J. Ecol. Eng..

[cit54] Zhang C., Li H., Du B. (2024). BioResources.

[cit55] Adam M., Mohd D., Ghani Z. A. (2025). J. Chem..

