# Supplementary material for: Cellulose and wood derived activated carbons from Pinus sylvestris: comparative performance in methylene blue adsorption
Source: RSC Adv. 2025 Dec 12;15(58):49780–95. doi: 10.1039/d5ra08022c (PMC12700138; doi:10.1039/d5ra08022c)

Supplementary Information (SI) for RSC Advances.
This journal is © The Royal Society of Chemistry 2025

Supporting Information

Title: Cellulose and Wood Derived Activated Carbons from Pinus sylvestris: Comparative Performance in Methylene Blue Adsorption

Authors: Nadia Anter [a]*, Abdellah Hannioui [a], Abdelouahid Medaghri-Alaoui [a], Mohssine Ghazoui [b]

***Correspondence to: nadia.anter@usms.ma**

Table of Contents

1. Cellulose extraction

2. Preparation of activated carbon

3. Comparison of the preparation conditions of AC-RW and AC-CRW

4. Adsorption isotherms

5. Comparison of adsorption efficiency (%) of MB between AC-RW and AC-CRW

6. Thermogravimetric analysis (TG-DTG).

7. SEM and BET of prepared AC-RW and AC- CRW

8. Characterization by FTIR spectrophotometry

9. X-ray diffraction analysis.

10. pH point zero charge of AC-RW and AC-CR

11. Effect of pH on adsorption

12. Effect of temperature on MB adsorption performance

13. Effect of contact time and dye concentration on adsorption

14. Adsorption kinetic

15. Adsorption isotherms

16. Thermodynamic study

17. Adsorption Mechanism of MB on AC-RW and AC-CRW

18. Cyclic Stability and Regeneration Efficiency of Activated Carbons

1. Cellulose extraction

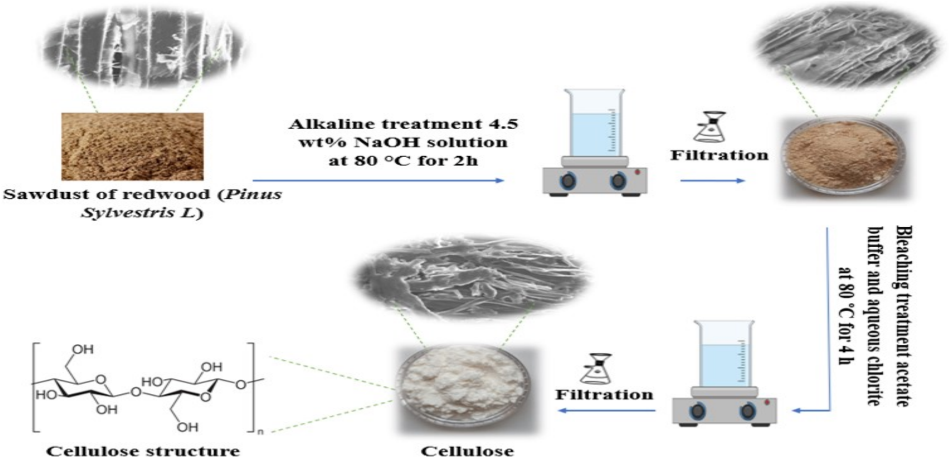

Fig. 1 Schematic representation of the cellulose isolation process from redwood (*Pinus sylvestris* L.) sawdust (*RW).*

2. Preparation of activated carbon

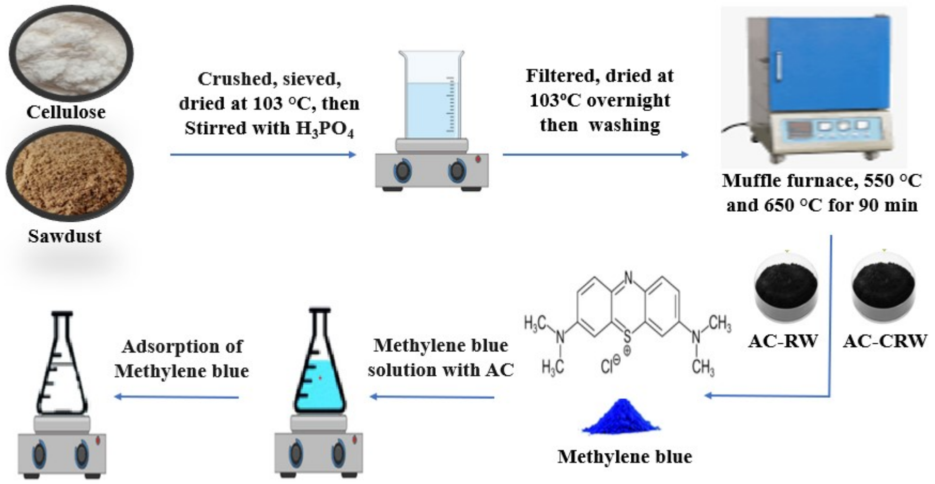

Fig. 2 Schematic illustration of the used process for activated carbons preparation and valorization as methylene blue adsorbents.

3. Comparison of the preparation conditions of AC-RW and AC-CRW

Table 1 Preparation conditions of AC-RW and AC-CRW: Type, Impregnation Time, Ratio, and Temperature.

Type of AC	Impregnation time (h)	Ratio	Temperature (°C)
**AC-RW**	16	1:1	550
**AC-CRW**	24	1:2	650

4. Adsorption isotherms

Table 2 Non-linear adsorption isotherm models and their characteristic parameters.

Isotherm Model	Non-linear Equation	Parameters	Ref.
Langmuir	$q_e = \frac{q_m K_L C_e}{1 + K_L C_e}$	q_{max}: maximum adsorption (mg/g) capacity; K_L: Langmuir constant	19,36
Freundlich	$q_e = K_F C_e^{1/n}$	K_F: Freundlich constant; n: adsorption intensity	37,38
Temkin	$q_e = Bln(K_T C_e)$	B: Temkin constant related to heat of adsorption; K_T: Temkin isotherm constant	39
Sips	$q_e = \frac{q_m (K_s C_e)^n}{1 + (K_L C_e)^n}$	K_s : (L/mg) is the Sips equilibrium constant; n : Heterogeneity index	39,40

5. Comparison of adsorption efficiency (%) of MB between AC-RW and AC-CRW

Table 3 Adsorption efficiency (%) of MB on AC-RW and AC-CRW under different preparation conditions.

**Runs**	**Type of AC**	**Impregnation time (h)**	**Ratio**	**Temperature (°C)**	**Adsorption Efficiency (%)**
1	AC-RW	16	1:1	550	78
2	AC-RW	16	1:2	550	82
3	AC-RW	24	1:1	550	81
4	AC-RW	24	1:2	550	86
5	AC-RW	16	1:1	650	84
6	AC-RW	16	1:2	650	88
7	AC-RW	24	1:1	650	87
8	AC-RW	24	1:2	650	92
9	AC-CRW	16	1:1	550	83
10	AC-CRW	16	1:2	550	87
11	AC-CRW	24	1:1	550	86
12	AC-CRW	24	1:2	550	91
13	AC-CRW	16	1:1	650	89
14	AC-CRW	16	1:2	650	93
15	AC-CRW	24	1:1	650	91
16	AC-CRW	24	1:2	650	96

6. Thermogravimetric analysis (TG-DTG).

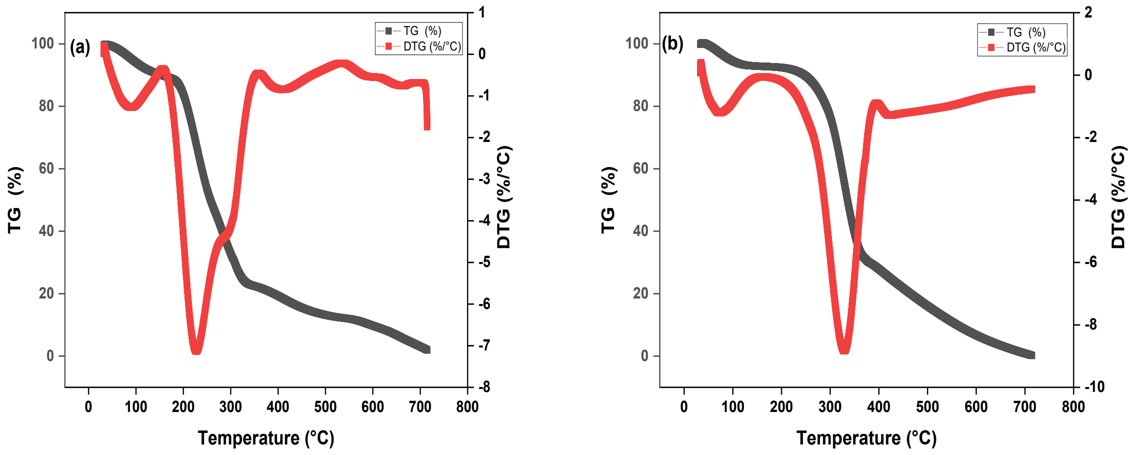

Fig. 3 TG/DTG of (a) RW (*Pinus Sylvestris L.*) and (b) cellulose.

7. SEM and BET of prepared AC-RW and AC- CRW

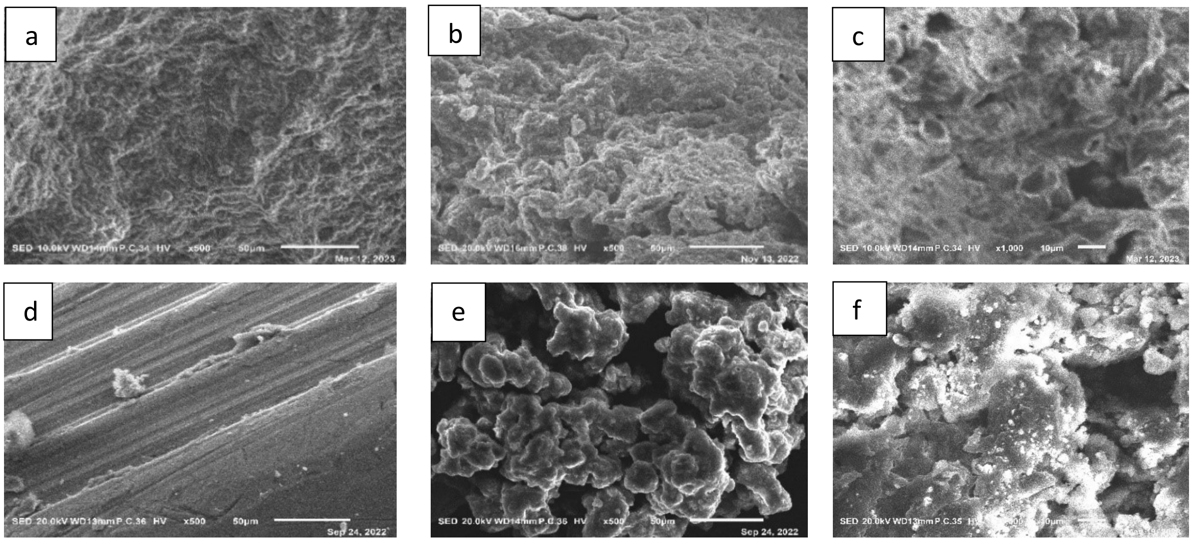

Fig. 4 SEM of optimal AC-RW (a) before activation and (b,c) after activation and AC-CRW (d) before activation and (e,f) after activation .

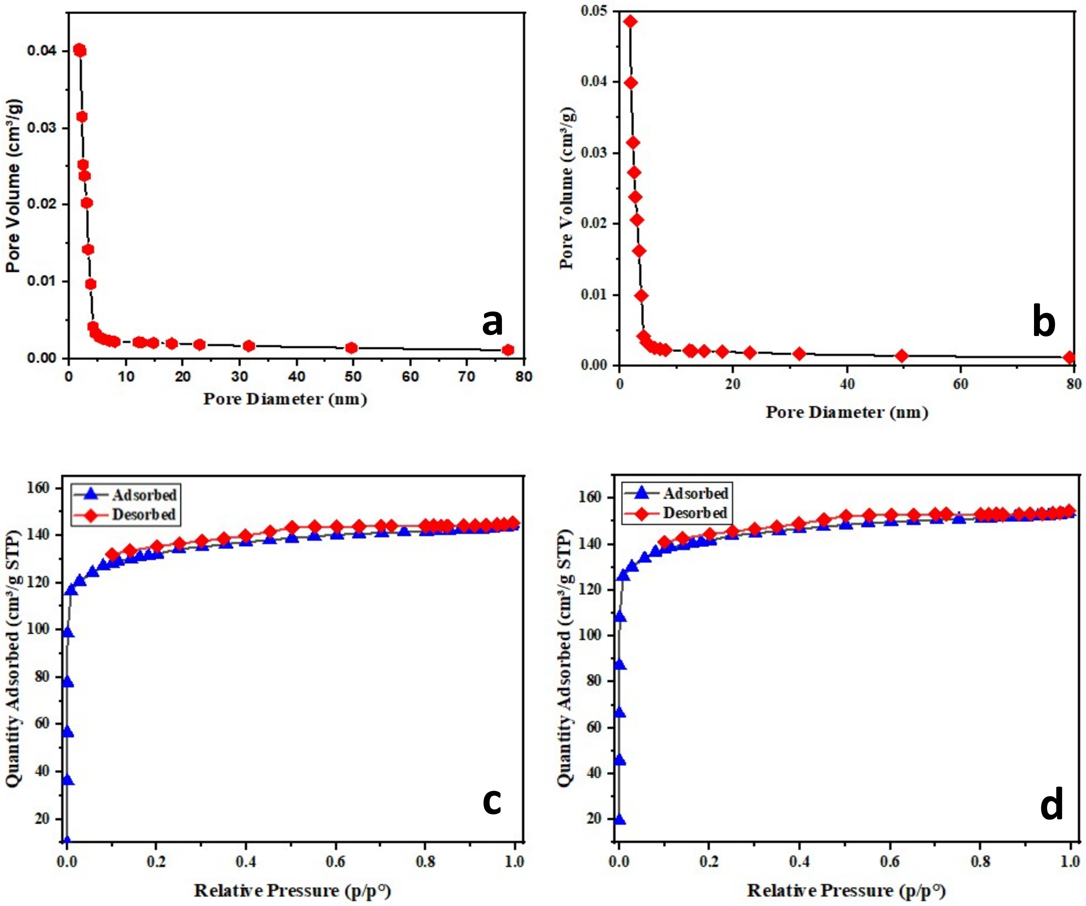

Fig. 5 N_2 adsorption-desorption isotherms and pore size distributions of AC-RW (a,c) and AC-CRW (b,d).

8. Characterization by FTIR spectrophotometry

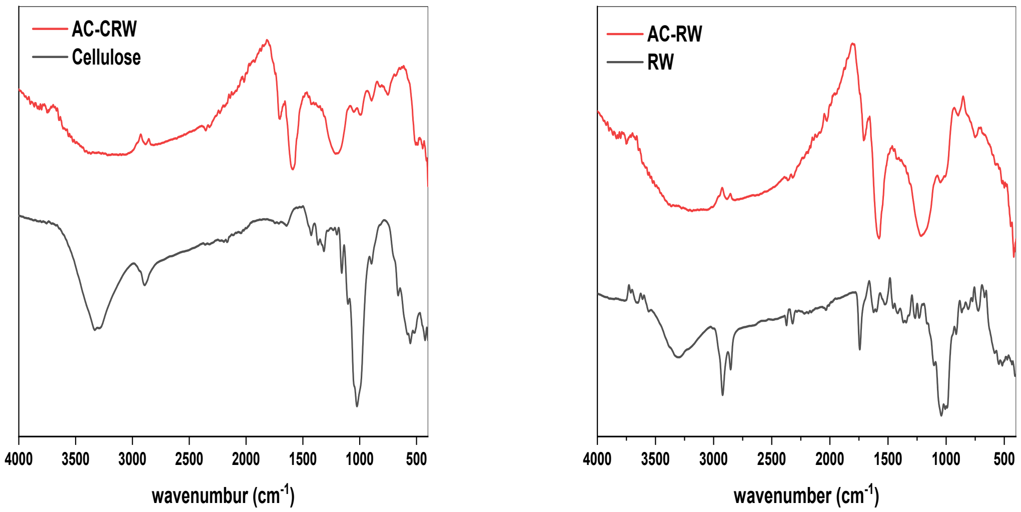

Fig. 6 FTIR spectra of RW, AC-RW, Cellulose and AC- CRW.

9. X-ray diffraction analysis.

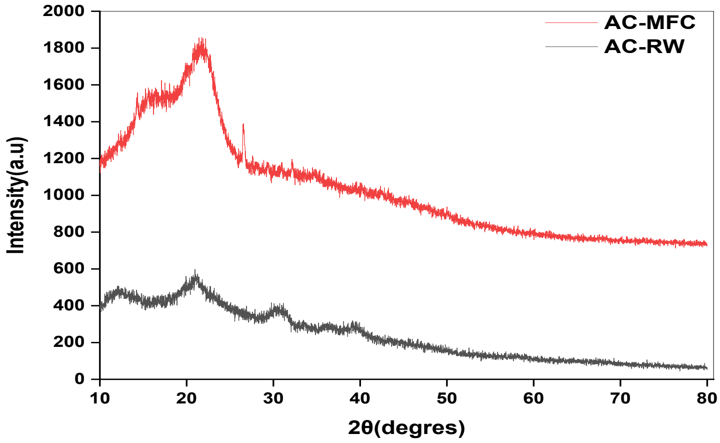

Fig. 7 XRD spectra of optimal *AC-RW and AC*-CRW.

10. pH point zero charge of AC-RW and AC-CR

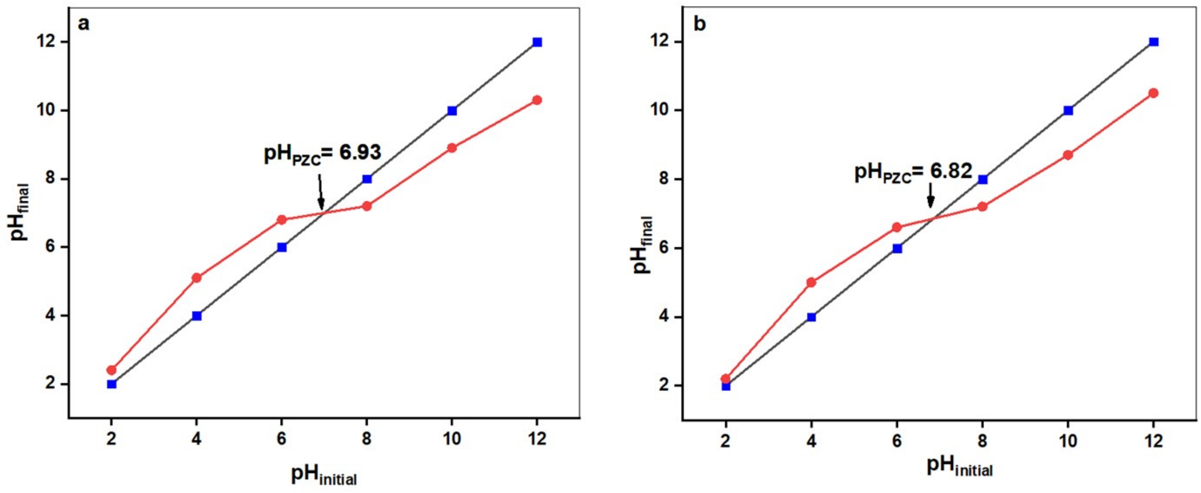

Fig.8 Determination of the point of zero charge pH_{PZC} for the prepared activated carbons: (a) cellulose-derived carbon (AC-CRW) and (b) wood-derived carbon (AC-RW).

11. Effect of pH on adsorption

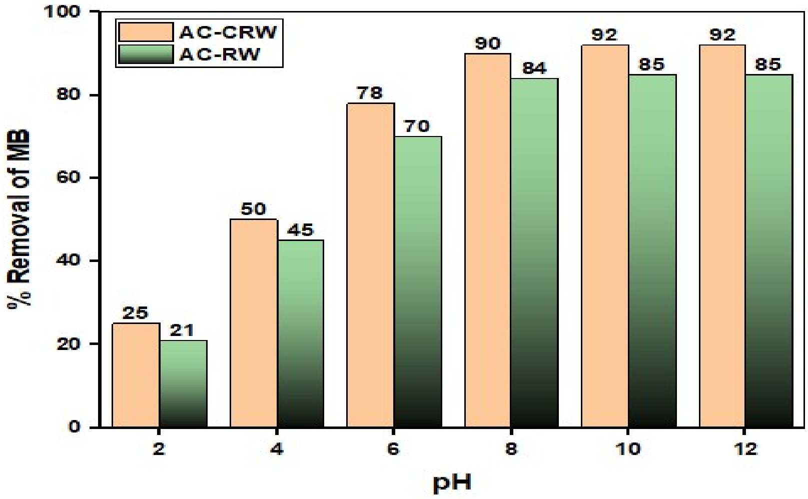

Fig. 9 Effect of solution pH on MB removal (%) by AC-CRW and AC-RW.

12. Effect of temperature on MB adsorption performance

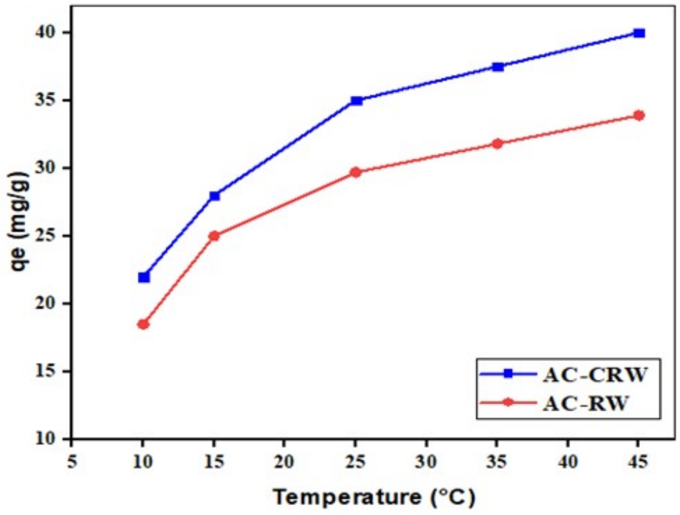

Fig. 10 Temperature effect on the adsorption capacity (qe) of MB onto AC-CRW and AC-RW (10–45 °C).

13. Effect of contact time and dye concentration on adsorption

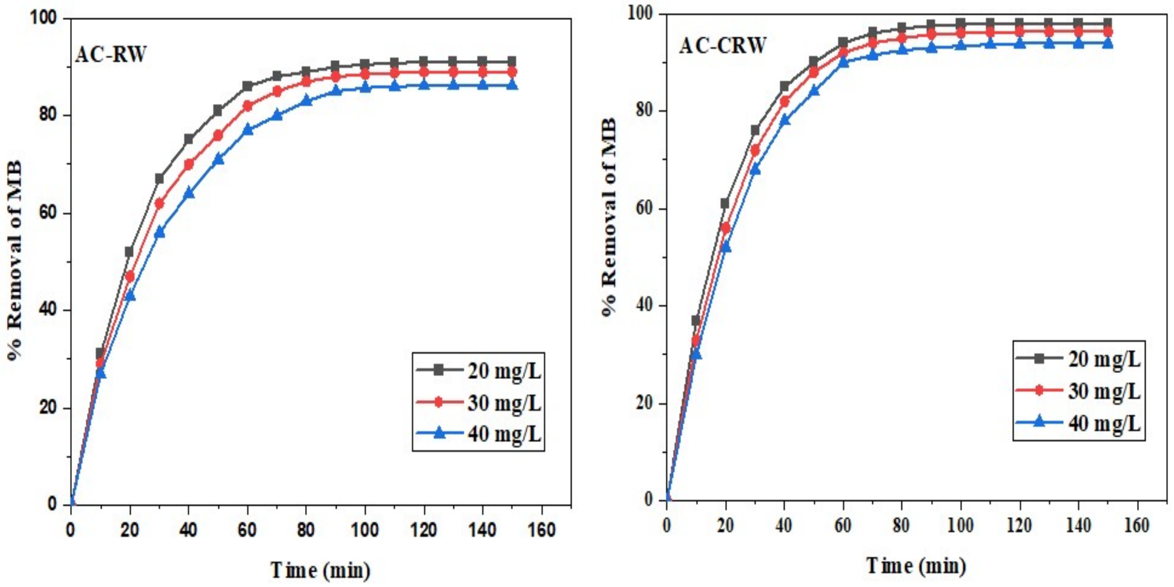

Fig. 11 Effect of contact time and initial concentration on the adsorption of methylene blue a) AC-RW and b) AC-CRW (conditions:100 mg of AC at.25 °C).

14. Adsorption kinetic

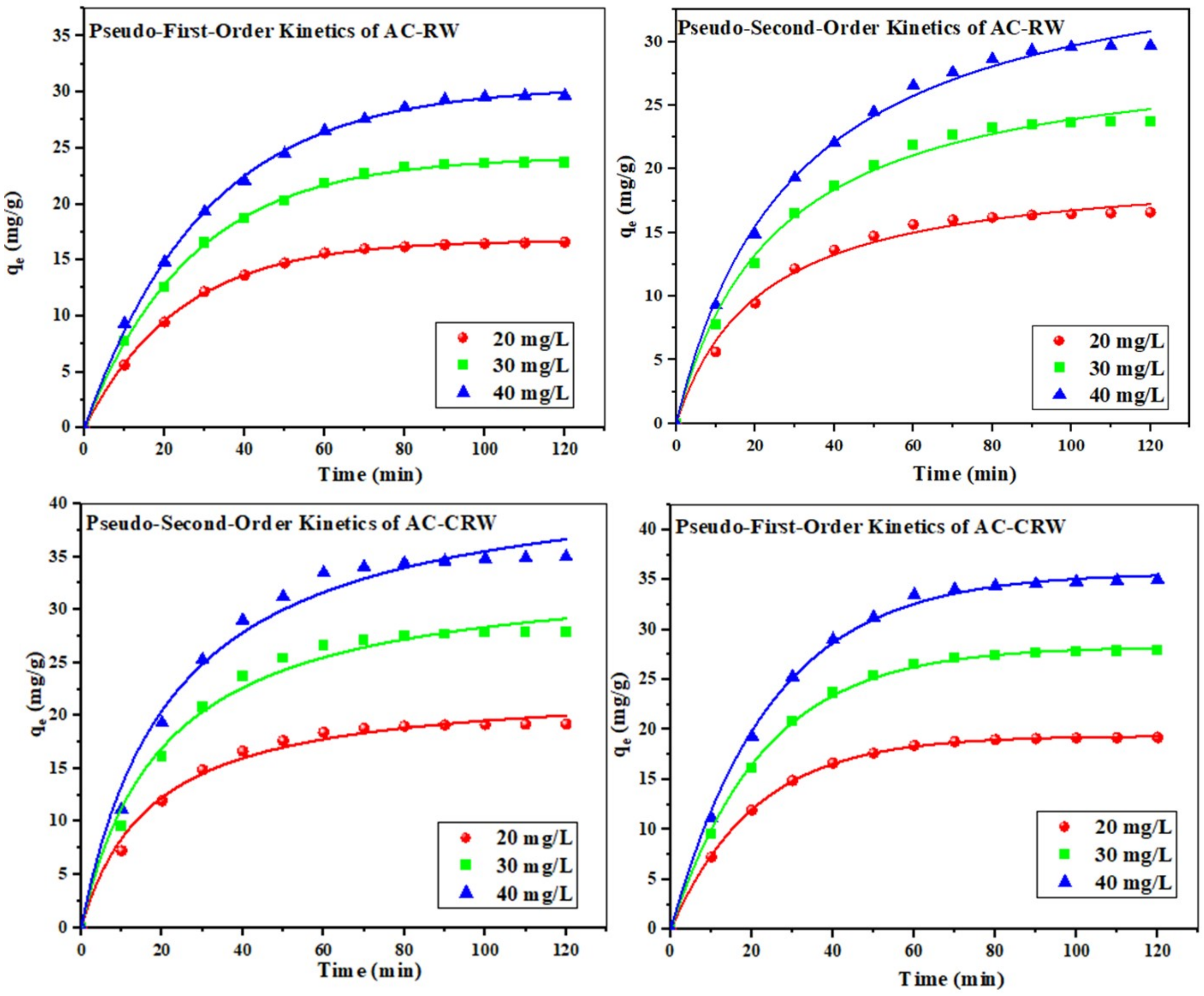

Fig. 12 Adsorption kinetics of MB onto AC-RW and AC-CRW at 25 °C fitted with pseudo-first-order and pseudo-second-order models.

Table 4 Kinetic parameters of pseudo-first-order and pseudo-second-order for MB using optimal AC-RW and AC-CRW.

**Kinetics model**	**Initial concentration (mg/L)**	**Kinetics constant**	**Type of adsorbent**	
			**AC-CRW**	**AC-RW**
First-order kinetic model40	20	$q_{e,exp}$ (mg/g)	19.21	16.6
		k_1 (min^{-1})	0.042	0.042
		$q_{e,cal}$ (mg/g)	19.37	16.77
		R^2	0.99978	0.99957
	30	$q_{e,exp}$ (mg/g)	27.9	23.7
		k_1 (min^{-1})	0.0126	0.037
		$q_{e,cal}$	28.31	24.21
		R^2	0.99909	0.99927
	40	$q_{e,exp}$ (mg/g)	35	29.7
		k_1 (min^{-1})	0.043	0.033
		$q_{e,cal}$	35.69	30.52
		R^2	0.99829	0.99899
Second-order kinetic model	20	k_2[g(mg^{-1}.min^{-1})]	0.0025	0.0023
		$q_{e,cal}$ (mg/g)	20.90	20.28
		R^2	0.98898	0.99045
	30	k_2[g(mg^{-1}.min^{-1})]	0.00145	0.00134
		$q_{e,cal}$ (mg/g)	34.07	29.85
		R^2	0.9869	0.99271
	40	k_2[g(mg^{-1}.min^{-1})]	0.0012	0.0088
		$q_{e,cal}$ (mg/g)	43.52	38.37
		R^2	0.98578	0.99587

15. Adsorption isotherms

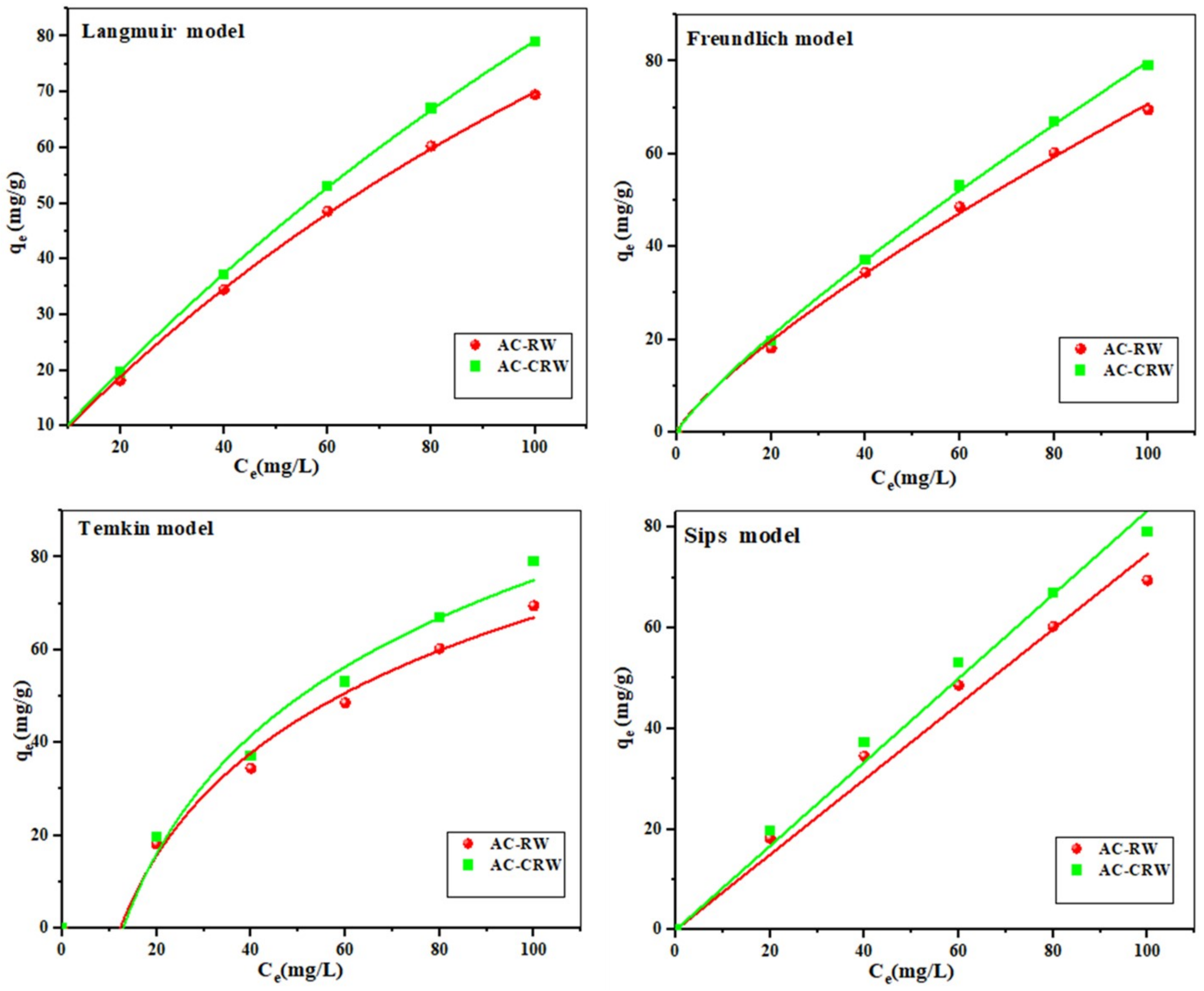

Fig. 13 Adsorption isotherm models *of MB by optimal AC-RW and AC-CRW.*

Table 5 Non-linear isotherm model parameters and statistical indicators for MB adsorption on optimal *AC-RW and AC-CRW at* 25°C.

Isotherm	Parameters	Type of adsorbent	
		**AC-RW**	**AC-CRW**
Langmuir	q_m (mg/g)	218.779	314.067
	K_L (L/mg)	0.0047	0.0033
	R^2 adjusted	0.99961	0.99993
	χ^2	0.266	0.062
Freundlich	K_F ((mg/g) (L/ mg)$^{1/n}$)	1.8477	1.69
	n	1.262	1.1941
	1/n	0.792	0.837
	R^2 adjusted	0.99744	0.99895
	χ^2	1.763	0.923
Temkin	B	31.865	36.598
	K_T(L.mg^{-1})	0.0818	0.0777
	R^2 adjusted	0.98985	0.98389
	χ^2	7.000	14.187
Spis	$K_{rp\ (mg/g)}$	0.747	0.833
	a	3×10^{-6}	0.0963
	g	-263.50	-221.88
	R^2 adjusted	0.96443	0.9803
	χ2	24.530	17.350

Table 6 Comparative study of the adsorption capacity of optimal AC-CRW and AC-RW for MB dye against different activated carbons.

Adsorbent	Time (min)	pH	T (°C)	qmax (mg/g)	Isotherm Model	Ref.
AC-CRW	70	7	25	314.06	Langmuir	This study
AC-RW	90	7	25	218.77	Langmuir	This study
Biomass-derived AC (general; chem-activation)	-	-	-	178.41	Langmuir	50
Spent coffee-ground carbon (acid/base-treated)	-	-	-	171.6 – 270.64	Langmuir	51
Raw olive-waste adsorbent (no high-T activation)	-	8	-	232.6 – 252.1	Langmuir	52
Hydrothermal carbon from palm kernel shell (KCS-2)	-	-	-	54.01	Langmuir	53
Cellulose-based activated carbon (CsCl)				176	Langmuir	54
Coffee-waste AC	60	6	25	176	Langmuir	55

16. Thermodynamic study

Table 7 Thermodynamic parameters for MB dye adsorption on AC-CRW and AC-RW adsorbent.

**Type of AC**	**ΔG° (KJ mol^{-1})**	**ΔH° (KJ mol^{-1})**	**ΔS° (Kj^{-1} mol^{-1})**

	283.15 K	288.15 K	298.15 K	308.5 K		
AC-CRW	−0.64	−1.94	−4.54	−7.14	72.63	259.94
AC-RW	−0.08	−0.82	−2.30	−3.77	41.75	147.80

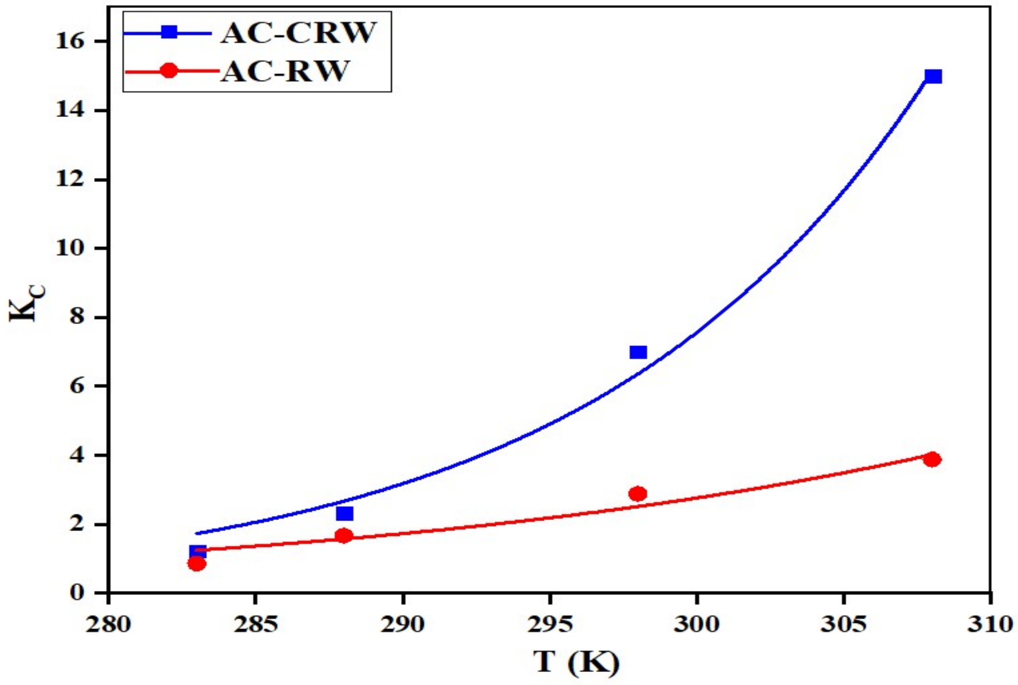

Fig. 14 Van't Hoff thermodynamic plot.

17. Adsorption Mechanism of MB on AC-RW and AC-CRW

Fig. 15 Proposed adsorption mechanism of MB onto AC-RW and AC-CRW.

18. Cyclic Stability and Regeneration Efficiency of Activated Carbons

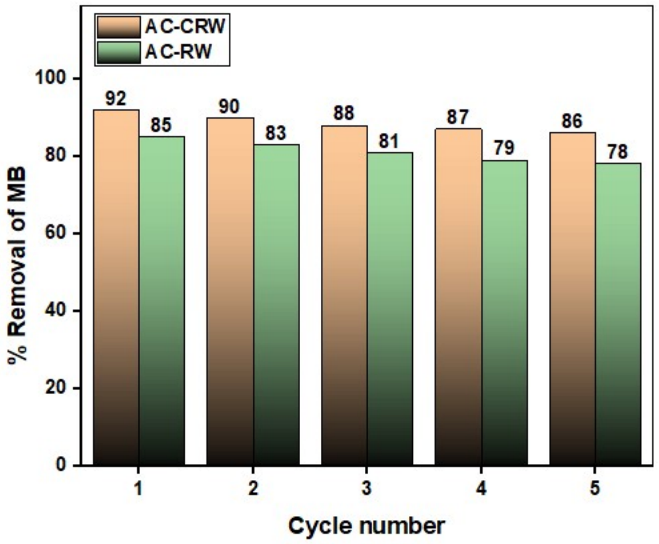

Fig. 16 Regeneration over five cycles: MB removal (%) by AC-CRW and AC-RW.

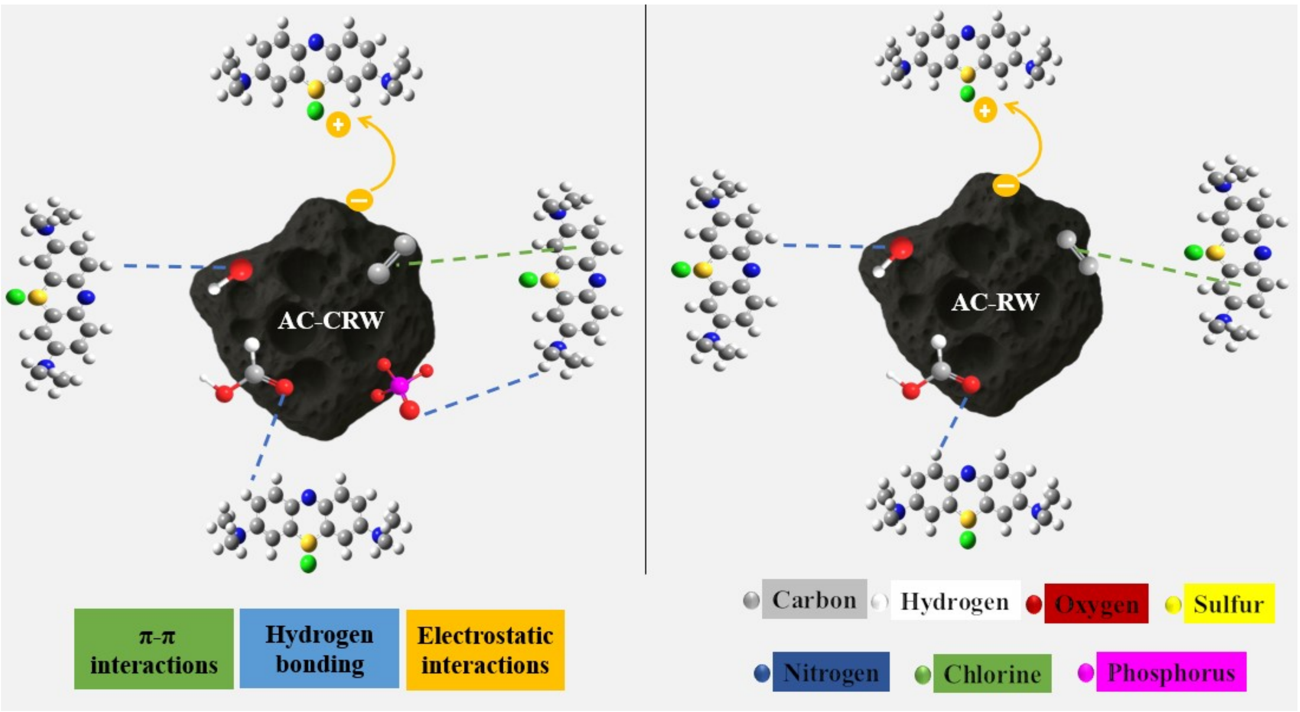

Supplement: RA-015-D5RA08022C-s001 [file RA-015-D5RA08022C-s001.pdf]
